# Multi-trait association studies discover pleiotropic loci between Alzheimer’s disease and cardiometabolic traits

**DOI:** 10.1186/s13195-021-00773-z

**Published:** 2021-02-04

**Authors:** William P. Bone, Katherine M. Siewert, Anupama Jha, Derek Klarin, Scott M. Damrauer, The VA Million Veteran Program, Zuhair K. Ballas, Sujata Bhushan, Edward J. Boyko, David M. Cohen, John Concato, Joseph I. Constans, Louis J. Dellitalia, Joseph M. Fayad, Ronald S. Fernando, Hermes J. Florez, Melinda A. Gaddy, Saib S. Gappy, Gretchen Gibson, Michael Godschalk, Jennifer A. Greco, Samir Gupta, Salvador Gutierrez, Kimberly D. Hammer, Mark B. Hamner, John B. Harley, Adriana M. Hung, Mostaqul Huq, Robin A. Hurley, Pran R. Iruvanti, Douglas J. Ivins, Frank J. Jacono, Darshana N. Jhala, Laurence S. Kaminsky, Scott Kinlay, Jon B. Klein, Suthat Liangpunsakul, Jack H. Lichy, Stephen M. Mastorides, Roy O. Mathew, Kristin M. Mattocks, Rachel McArdle, Paul N. Meyer, Laurence J. Meyer, Jonathan P. Moorman, Timothy R. Morgan, Maureen Murdoch, Xuan-Mai T. Nguyen, Olaoluwa O. Okusaga, Kris-Ann K. Oursler, Nora R. Ratcliffe, Michael I. Rauchman, R. Brooks Robey, George W. Ross, Richard J. Servatius, Satish C. Sharma, Scott E. Sherman, Elif Sonel, Peruvemba Sriram, Todd Stapley, Robert T. Striker, Neeraj Tandon, Gerardo Villareal, Agnes S. Wallbom, John M. Wells, Jeffrey C. Whittle, Mary A. Whooley, Junzhe Xu, Shing-Shing Yeh, Michaela Aslan, Jessica V. Brewer, Mary T. Brophy, Todd Connor, Dean P. Argyres, Nhan V. Do, Elizabeth R. Hauser, Donald E. Humphries, Luis E. Selva, Shahpoor Shayan, Brady Stephens, Stacey B. Whitbourne, Hongyu Zhao, Jennifer Moser, Jean C. Beckham, Jim L. Breeling, J. P. Casas Romero, Grant D. Huang, Rachel B. Ramoni, Sumitra Muralidhar, Samuel M. Aguayo, Sunil K. Ahuja, Saiju Pyarajan, Yan V. Sun, Kelly Cho, J. Michael Gaziano, Peter W. Wilson, Christopher J. O’Donnell, Kyong-Mi Chang, Philip S. Tsao, Themistocles L. Assimes, Marylyn D. Ritchie, Benjamin F. Voight

**Affiliations:** 1 Genomics and Computational Biology Graduate Group, Perelman School of Medicine, University of Pennsylvania, Philadelphia, PA 19104 USA; 2 Department of Computer and Information Science, School of Engineering and Applied Science, University of Pennsylvania, Philadelphia, PA 19104 USA; 3 Boston VA Healthcare System, Boston, MA 02130 USA; 4 Center for Genomic Medicine, Massachusetts General Hospital, Harvard Medical School, Boston, MA 02114 USA; 5 Program in Medical and Population Genetics, Broad Institute of MIT and Harvard, Cambridge, MA 02142 USA; 6 Department of Medicine, Perelman School of Medicine, University of Pennsylvania, PA 19104 Philadelphia, USA; 7 Corporal Michael Crescenz VA Medical Center, Philadelphia, PA 19104 USA; 8 Department of Surgery, Perelman School of Medicine, University of Pennsylvania, Philadelphia, PA 19104 USA; 9 VA Palo Alto Health Care System, Palo Alto, CA 94550 USA; 10 Department of Medicine, Stanford University School of Medicine, Stanford, CA 94305 USA; 11 Department of Genetics, Perelman School of Medicine, University of Pennsylvania, Philadelphia, PA 19104 USA; 12 Institute for Biomedical Informatics, Perelman School of Medicine, University of Pennsylvania, Philadelphia, PA 19104 USA; 13 Center for Precision Medicine, Perelman School of Medicine, University of Pennsylvania, Philadelphia, PA 19104 USA; 14 Department of Systems Pharmacology and Translational Therapeutics, Perelman School of Medicine, University of Pennsylvania, Philadelphia, PA 19104 USA; 15 Institute for Translational Medicine and Therapeutics, Perelman School of Medicine, University of Pennsylvania, Philadelphia, PA 19104 USA

**Keywords:** Pleiotropy, Cardiometabolic traits, Multi-trait GWAS, Colocalization

## Abstract

**Background:**

Identification of genetic risk factors that are shared between Alzheimer’s disease (AD) and other traits, i.e., pleiotropy, can help improve our understanding of the etiology of AD and potentially detect new therapeutic targets. Previous epidemiological correlations observed between cardiometabolic traits and AD led us to assess the pleiotropy between these traits.

**Methods:**

We performed a set of bivariate genome-wide association studies coupled with colocalization analysis to identify loci that are shared between AD and eleven cardiometabolic traits. For each of these loci, we performed colocalization with Genotype-Tissue Expression (GTEx) project expression quantitative trait loci (eQTL) to identify candidate causal genes.

**Results:**

We identified three previously unreported pleiotropic trait associations at known AD loci as well as four novel pleiotropic loci. One associated locus was tagged by a low-frequency coding variant in the gene *DOCK4* and is potentially implicated in its alternative splicing. Colocalization with GTEx eQTL data identified additional candidate genes for the loci we detected, including *ACE*, the target of the hypertensive drug class of ACE inhibitors. We found that the allele associated with decreased *ACE* expression in brain tissue was also associated with increased risk of AD, providing human genetic evidence of a potential increase in AD risk from use of an established anti-hypertensive therapeutic.

**Conclusion:**

Our results support a complex genetic relationship between AD and these cardiometabolic traits, and the candidate causal genes identified suggest that blood pressure and immune response play a role in the pleiotropy between these traits.

**Supplementary Information:**

The online version contains supplementary material available at 10.1186/s13195-021-00773-z.

## Background

Studies have consistently found a positive epidemiological correlation between Alzheimer’s disease (AD) and cardiometabolic traits, yet the biological mechanisms behind this correlation is not well understood [1–4]. A leading hypothesis is that this correlation is due to shared genetic influence, or pleiotropy, between AD and cardiometabolic traits [4]. By identifying pleiotropic loci between these traits, we can (i) identify new therapeutic targets or opportunities for drug repurposing, (ii) predict potential side effects, and (iii) better understand the etiology of these complex traits. The identification of new therapeutic targets for AD is of particular importance since AD afflicts approximately 50 million people, and there exist only a handful of therapeutics available for AD that have only limited efficacy in slowing the progression of the disease [5].

Pleiotropy has been an area of both theoretical and empirical study at least since the beginning of the twentieth century [6–8]. However, the topic has received renewed attention, given the pervasiveness of pleiotropy that has been uncovered through genome-wide association studies (GWAS) [8–10]. Recent methods and analysis have sought to characterize the extent of the phenomenon throughout the genome [8], quantifying pairwise genetic correlation across a battery of traits [8, 11], exploiting pleiotropy to perform causal inference in the framework of Mendelian randomization [8, 12], or statistically co-localizing association signals across two or more traits [13, 14]. These methods and publicly available GWAS summary statistics enable studies to dissect the shared genetic etiology between AD and cardiometabolic traits. Due to the epidemiological correlation between AD and cardiometabolic traits, coupled with the fact that many cardiometabolic traits are genetically correlated with one another, additional broader-scale pleiotropic studies are warranted, and recently the field has begun to do so [4, 11].

Statistical methods for detecting pleiotropy use the definition of a single locus associated with two or more traits, and these methods are generally intended to detect loci that have a single genetic variant underlying the shared heritability at the locus. However, recent studies have shown that at some pleiotropic loci there is no shared causal SNP, but instead different SNPs are causal for the different traits. These loci are associated with multiple traits but there is no shared causal genetic variant behind the associations [9, 15]. For this reason, we consider here a more stringent definition of pleiotropy: loci that are associated with two or more traits, *and* where the statistical data provides evidence of a shared causal genetic variant. We used colocalization analysis to identify which loci appear to share causal genetic variants and which appear to be cases of spurious pleiotropy [8, 13]. There are two models of pleiotropy for this scenario [8]. The first is horizontal pleiotropy, where a genetic variant has a direct effect on two or more traits. The other is vertical pleiotropy, where a genetic variant has a direct effect on a trait and a mediated effect on a second trait through the first trait [8].

In this study, we used summary statistics from the largest publicly available single-trait GWAS to investigate pleiotropy between AD and eleven cardiometabolic traits using the metaMANOVA bivariate GWAS method followed by colocalization analysis [16]. This bivariate GWAS method takes summary statistics for two traits as input and performs a GWAS for the pair of traits, while taking the correlation across association statistics into account [16]. We used this method to perform two different experiments. The first experiment was an “AD-centric” analysis, intended to detect loci that are associated with AD, but previously not shown to be pleiotropic for cardiometabolic traits. We also performed a locus discovery analysis to discover loci that are not previously reported to be associated with either AD or the cardiometabolic trait.

## Methods

We performed two bivariate GWAS experiments intended to detect loci that are pleiotropic between AD and cardiometabolic traits. For ease of reproducibility, we first performed a pairwise bivariate GWAS between AD and each of eleven cardiometabolic traits for both experiments. We then assessed whether there was evidence of a shared causal SNP at each bivariate significant locus by performing a colocalization analysis between the AD and cardiometabolic trait signals. To identify candidate causal genes, we performed colocalization analyses between the pleiotropic signals and single-tissue eQTLs from Genotype-Tissue Expression (GTEx) project v7 [13].

### Bivariate GWAS

We used the summary statistics from publicly available single-trait GWAS to perform pairwise metaMANOVA bivariate GWAS between AD [17] and the following cardiometabolic traits: coronary heart disease (CHD) [18], type II diabetes (T2D) [19], systolic blood pressure (SBP) [20], diastolic blood pressure (DBP) [20], body mass index (BMI) [21], waist-hip ratio adjusted for BMI (WHRadjBMI) [22], body fat percentage (BFP) [23], total cholesterol (TC) [24], low-density lipoproteins (LDL) [24], high-density lipoproteins (HDL) [24], and triglycerides (TG) [24] (Table 1 and Additional file 1 - Supplementary Table 1; Availability of data and materials). For each of these studies, approval by an institutional review committee was obtained, and all subjects gave informed consent, as documented in each original publication. All bivariate GWAS were performed using the *bivariate_scan* software [16]. Each bivariate GWAS resulted in a set of independent loci, which we defined as the genomic region that includes all SNPs within 1 MB of the bivariate lead SNP and any other SNPs that are in LD of *r*^2^ > 0.2 with the lead SNP using the 1000 Genomes European ancestry cohort (1 kG EUR) [25]. Further detail on our bivariate GWAS pipeline can be found in the Additional file 1 - Supplemental Methods [16].

**Table 1:** Single-trait GWAS summary statistics used for bivariate GWAS

Trait	Publication	PMID	Sample size
AD	Jansen et al. [17]	30617256	71,880 cases, 383,378 controls
BFP	Lu et al. [23]	26833246	100,716
BMI	Yengo et al. [21]	30124842	681,275
CHD	Van der Harst et al. [18]	29212778	34,541 cases, 261,984 controls
DBP	Evangelou et al. [20]	30224653	1,006,863
HDL	Klarin et al. [24] (EUR samples only)	30275531	404,128
LDL	Klarin et al. [24] (EUR samples only)	30275531	404,128
SBP	Evangelou et al. [20]	30224653	1,006,863
TC	Klarin et al. [24] (EUR samples only)	30275531	404,128
TG	Klarin et al. [24] (EUR samples only)	30275531	404,128
T2D	Mahajan et al. [19]	30297969	80,831 cases, 817,299 controls
WHRadjBMI	Pulit et al. [22]	30239722	694,649

A list of the traits, original GWAS publication, and sample sizes for each trait used in our analyses

### AD-centric analysis

**Fig. 1:**
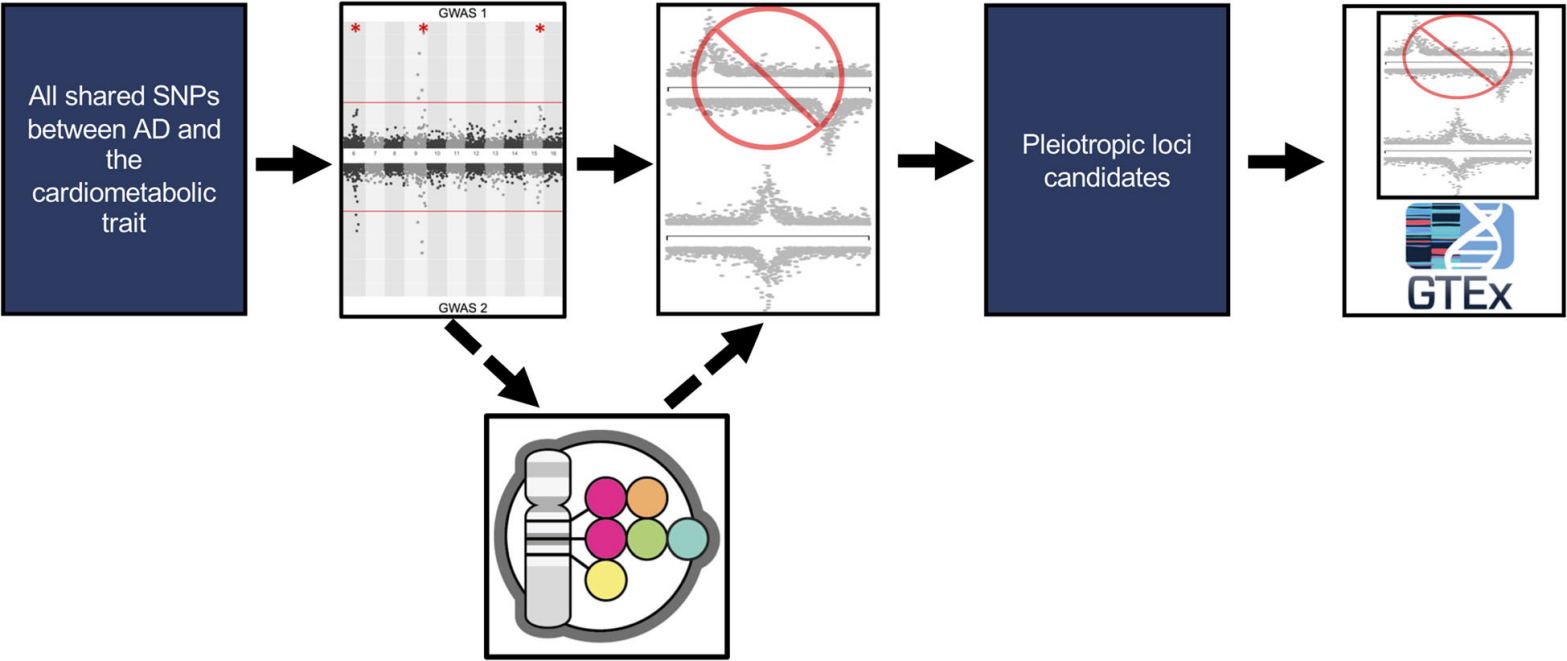
Bivariate GWAS analysis workflow. Starting with all the SNPs that were in both GWAS summary statistics files, we performed a bivariate GWAS and filtered the bivariate significant loci based on their single-trait *P* values. For the locus discovery experiment, we removed loci that were in LD (1 kG EUR *r*^2^ > 0.2) or within 500 kb of a known AD or the cardiometabolic trait being tested according to the GWAS Catalog (dotted line arrows). The filtration steps were followed by trait-trait colocalization to confirm there was evidence of a shared causal SNP between the signals at each locus. Finally, we performed single-tissue-eQTL analysis to identify candidate causal genes for each locus

We performed an AD-centric analysis to identify loci that are known to be associated with AD, but not previously known to be pleiotropic for cardiometabolic traits. We first performed pairwise bivariate GWAS between AD and each cardiometabolic trait (Additional file 1 - Supplementary Table 2). To reduce the list of bivariate GWAS genome-wide significant loci results to just the loci that are near genome-wide significantly associated with AD and potentially associated with a cardiometabolic trait, we applied a filter that required loci to have an AD *P* value < 1 × 10^− 6^ and a cardiometabolic trait *P* value < 5 × 10^− 3^ (Fig. 1).

### Locus discovery analysis

To performed a locus discovery analysis, we performed a bivariate GWAS between AD and each cardiometabolic trait (Additional file 1 - Supplementary Table 3). To identify loci that were both pleiotropic and novel, we required the bivariate GWAS lead SNP had *r*^2^ < 0.2 in 1 kG EUR and was greater than 500 kb away from all known single-trait associated loci for AD or the cardiometabolic trait being tested, as well as any loci from previous pleiotropic GWAS between the two traits [4, 26]. Additionally, each locus needed to have at least a nominal single-trait association with both traits, so we required an AD *P* value < 5 × 10^− 3^ and a cardiometabolic trait *P* value < 5 × 10^− 3^ (Fig. 1).

### Trait-trait colocalization

We performed colocalization analysis between the AD and the cardiometabolic trait signals given a 500 kb window (±  250 kb) around each locus using *COLOC* [13]. Our threshold for this analysis was a conditional probability of colocalization (i.e., PP4/ (PP3 + PP4)) ≥ 0.8, which is defined as the posterior probability of colocalization conditioned on the presence of a signal for each trait (Fig. 1). Loci that had a conditional probability of colocalization > 0.45 and < 0.8 were visually inspected using *LocusZoom* plots, and if the LD structure suggested additional associations unlinked to the leading variant in the region, we performed approximate conditional analysis (see “Approximate conditional analysis,” below) [27]. We excluded loci in the *HLA* region and near the *APOE* locus from these experiments due to the difficulty in interpreting the independent contribution of these loci to these traits.

### Single-tissue-eQTL colocalization

We performed single-tissue eQTL colocalization analysis to prioritize candidate causal genes implicated by the pleiotropic signals detected in our bivariate GWAS. We collected the list of genes and tissues for which each bivariate GWAS lead SNP was a significant single-tissue eQTL in GTEx v7 from the GTExPortal (Additional file 1 - Supplementary Tables 4–7,9; Additional file 2 -Supplementary Table 8) (data from GTEx as of 02-28-2018, v7) [28]. We then performed colocalization using the AD association data at each locus and each single-tissue eQTL signal from GTEx v7 using a 500-kb window (± 250 kb) around the lead SNP using *COLOC* [13] (Fig. 1). As above, we considered the AD and eQTL signals to colocalize if the conditional probability of colocalization was ≥ 0.8. We visually inspected the loci where the colocalization analysis resulted in a standard probability of colocalization < 0.8, but conditional probability of colocalization met our criteria [27]. For these loci, we performed approximate conditional analysis, when the LD structure suggested there could be allelic series (see “Approximate conditional analysis,” below).

### Approximate conditional analysis

At each locus, we performed approximate conditional analysis on SNPs that appeared to be associated with the trait of interest independently of the lead SNP, because the presence of multiple associated variants in a region violates the assumptions of *COLOC* and can lead to false positives or false negatives [13]. We identified potential nearby association signals using *LocusZoom* plots and the *LDassoc* tool of *LDlink* [27, 28]. For each locus, we performed approximate conditional analysis using *GCTA-COJO* with 1000 Genome Project data (European samples, *n* = 503) as a reference panel [29, 30]. We conditioned our lead SNP on the most associated SNP for each potential confounding signals we identified at the locus. We then repeated the colocalization experiment on the locus using the conditional SNP *P* values. We provide a full list of traits and loci we performed conditional analysis on, the lead SNP for each analysis, and the SNPs we conditioned on for each analysis are in the supplement (Additional file 1 - Supplementary Table 10).

## Results

### AD-centric analysis results

We performed an AD-centric analysis to detect known AD loci that were not previously known to be pleiotropic with eleven cardiometabolic traits (“Methods”). We identified a total of 39 independent loci that were bivariate genome-wide significant, met our AD-centric single-trait *P* value threshold of *P* value < 1 × 10^− 6^ and a cardiometabolic trait *P* value < 5 × 10^− 3^, and were outside of the *HLA* and *APOE* regions (Additional file 1 Supplementary Table 2).

We next performed trait-trait colocalization analysis on all 39 bivariate genome-wide significant loci to identify the subset of loci with evidence of a causal SNP shared in common between the AD signal and the cardiometabolic trait signal. Three loci met our colocalization criteria (Table 2). All of these loci are novel pleiotropic loci between AD and the respective cardiometabolic traits, but have previously been identified as genome-wide significant for AD in recent single-trait AD GWAS [4, 17, 31].

**Table 2:** AD-centric analysis pleiotropic loci

Cardiometabolic trait	Locus name	Lead SNP	Chr	Position GRCh37	Effect allele/other allele	Direction of effect AD/CM	Effect allele frequency	Bivariate *P* value	AD *P* value	Cardiometabolic trait *P* value	Conditional posterior probability of colocalization	Cardiometabolic GWAS
DBP	*ADAM10*	rs442495	15	59022615	T/C	+/+	0.65	1.98e−10	1.31e−09	9.71e−05	0.88	Evangelou et al. [20]
WHRadjBMI	*ADAMTS4*	rs4575098	1	161155392	A/G	+/+	0.22	4.45e−13	2.05e−10	1.99e−06	0.87	Pulit et al. [22]
DBP	*ACE*	rs4308	17	61559625	G/A	+/−	0.63	7.05e−15	8.52e−07	1.00e−16	0.98	Evangelou et al. [20]
SBP		rs4308	17	61559625	G/A	+/−	0.63	8.01e−15	8.52e−07	1.00e−16	0.98	Evangelou et al. [20]

*Chr* chromosome of the SNP. Direction of effect first position is the direction of effect of the effect allele on AD and the cardiometabolic trait. Effect allele frequency from the Jansen et al. [17] allele frequency. Conditional posterior probability of colocalization, PP4/ (PP3 + PP4) the results of the trait-trait colocalization analysis

To identify candidate causal genes at these three loci, we performed single-tissue-eQTL colocalization analysis between the AD signal at each locus using eQTLs identified by GTEx (“Methods”). All three pleiotropic signals colocalized with one or more single-tissue eQTL signals (Additional file 1 -Supplementary Table 11), and we describe these loci in more detail below.

We detected a pleiotropic signal between AD and DBP at the *ADAM10* locus, discovered as an AD association in Jansen et al. [17] (Additional file 1 - Fig. S1). Previous single-trait GWAS have identified several other cardiometabolic trait associations, including BMI and CHD, near this locus (within a 1-Mb window around the lead SNP), but our colocalization results suggest that these signals are independent of the AD signal at this locus [26]. Single-tissue eQTL colocalization analysis identified a single eQTL for *MINDY2* in tibial nerve tissue that met our colocalization threshold (Additional file 1 - Fig. S1 and Supplementary Table 11).

The second pleiotropic signal we detected was at the *ADAMTS4* locus between WHRadjBMI and AD, also discovered in Jansen et al. [17] AD GWAS (Additional file 1 - Fig. S2). Single-tissue-eQTL colocalization analysis demonstrated that eQTLs for the gene *NDUFS2* across multiple tissues strongly colocalized with this signal (Additional file 1 - Supplementary Table 11). An eQTL for the gene *FCER1G* in tibial nerve also met our colocalization threshold (Additional file 1 - Supplementary Table 11).

**Fig. 2:**
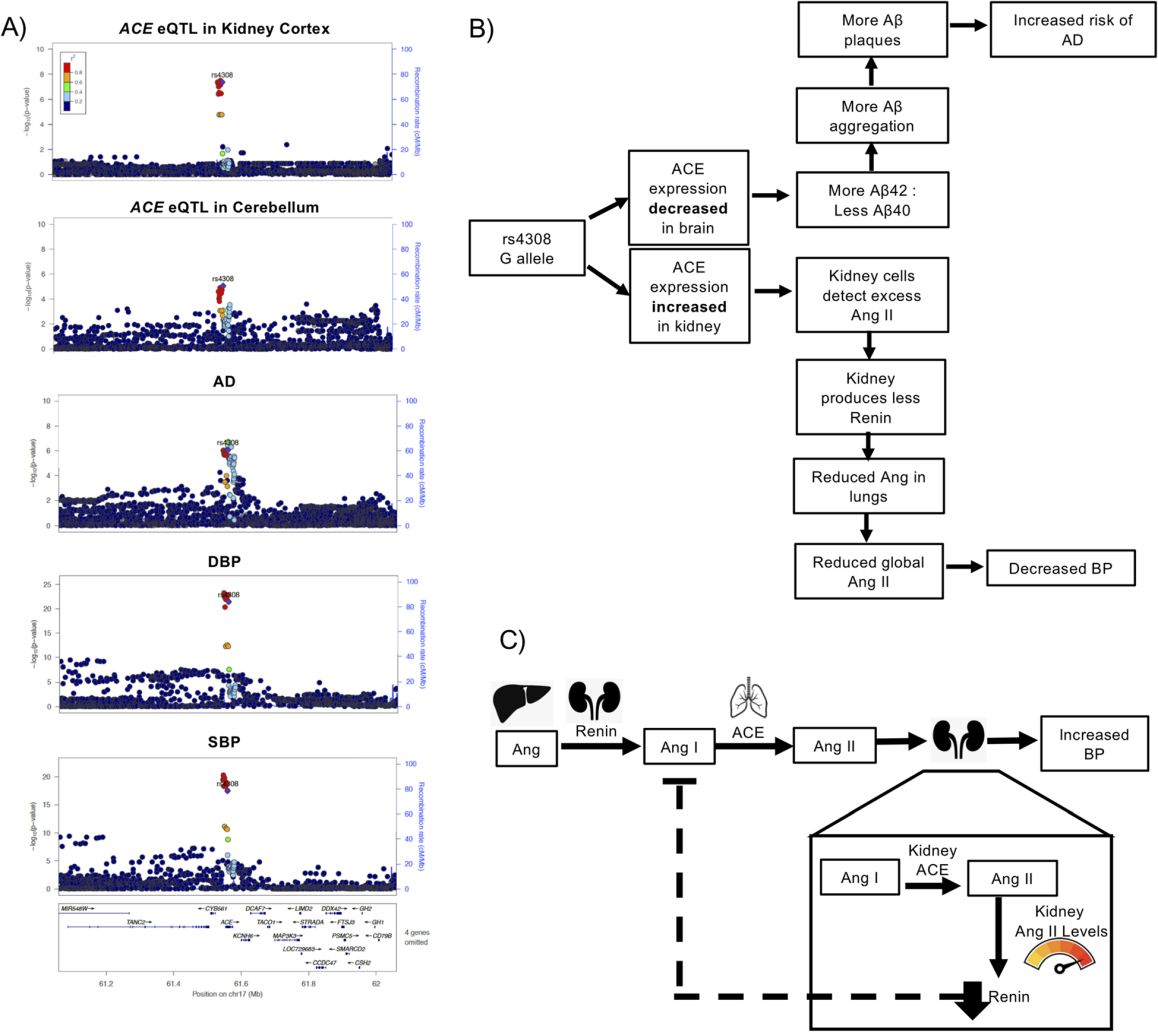
*ACE* locus. **a** Pleiotropic signal between DBP, SBP, and AD at the *ACE* locus, and the eQTL signal for *ACE* in kidney cortex and cerebellum. **b** Flowchart of our hypothesized mechanism as to how tissue-specific expression of *ACE* could mediate the blood pressure (BP) and AD pleiotropic signal at this locus. **c** Diagram of a hypothesized mechanism by which increased kidney expression of *ACE* could alter renin expression and thus lead to reduce BP through the feedback loops of the renin-angiotensin system

Finally, we detected pleiotropic signals at the *ACE* locus, which is a known blood pressure and AD association, between both DBP and AD and SBP and AD (Fig. 2a and Tables 2, [20, 31–35]). We noted a direction of effect opposite to the epidemiological correlation for both of these signals, meaning the allele that was associated with reduced risk of AD was associated with increased blood pressure. Our single-tissue eQTL colocalization showed that both pleiotropic signals had strong evidence of colocalization with eQTLs for *ACE* (Additional file 1 - Supplementary Table 11), but also were opposite directions of effect among different tissues (Additional file 1 - Supplementary Table 11, [36]).

The observed complexity of opposite direction effects at this locus motivated us to further investigate the potential of multiple variants associating with traits and/or eQTLs in the region to confound our colocalization analyses. Here, we performed approximate conditional analyses on the pleiotropic signal lead SNP, rs4308, and the lung *ACE* eQTL lead SNP, rs4324, in the single-tissue *ACE* eQTL data for kidney cortex (GTEx v8), lung (GTEx v7), and cerebellum (GTEx v7) (Table 3). The results of this analysis suggested that the *ACE* eQTL in lung was independent of the *ACE* eQTLs in the other tissues. These results also support that the *ACE* eQTLs in kidney and cerebellum share the same causal SNP, which has opposite directions of effect in these tissues (Table 3). Previous studies at this locus observed this same relationship between *ACE* expression in brain tissue and *ACE* expression in plasma [33].

**Table 3:** Approximate conditional analysis on tissue-specific allelic series in ACE eQTLs

Tissue of *ACE* eQTL	*P* value of rs4308 conditioned on rs4324	Effect of rs4308 on the ACE eQTL conditioned on rs4324	*P* value of rs4324 conditioned on rs4308	Effect of rs4324 on the ACE eQTL conditioned on rs4308
Lung	0.15	0.048	2.57e−08	0.18
Cerebellum	5.34e−04	− 0.25	0.66	− 0.034
Kidney cortex	4.41e−08	0.49	0.21	− 0.14

Approximate conditional analyses performed on the leading single-tissue eQTLs at the *ACE* locus

We next assessed which *ACE* eQTLs were most likely to be involved with each of the single-trait signals at this locus, which included the AD, DBP, and SBP signals that we report as pleiotropic as well as a T2D signal that occurred in this region (Additional file 1 - Fig. S3). We performed colocalization analysis of each of the trait signals with the single-tissue *ACE* eQTLs in kidney cortex, lung, and cerebellum (Table 4). The T2D signal colocalized with the lung *ACE* eQTL, but not with the kidney and cerebellum *ACE* eQTLs. The DBP and SBP signals colocalized with the cerebellum and kidney *ACE* eQTLs, but not the lung *ACE* eQTL. The AD signal colocalized with all three *ACE* eQTLs, but the evidence for colocalization was stronger for the cerebellum and kidney *ACE* eQTLs (Table 4). These results suggest that the blood pressure and AD pleiotropic signals share the same causal SNP that is in high LD with rs4308 and that these associations could be mediated by changes in *ACE* expression in kidney and brain tissue. However, the T2D signal at this locus appears to be independent of the rs4308 signal and could be mediated by changes in *ACE* expression in lung tissue.

**Table 4:** Tissue-specific *ACE* eQTL colocalization with GWAS trait signals at the *ACE* locus

Tissue of *ACE* eQTL	Conditional probability of colocalization with AD	Conditional probability of colocalization with T2D	Conditional probability of colocalization with DBP	Conditional probability of colocalization with SBP
Lung	0.89	0.96	2.73e−04	2.72e−04
Cerebellum	0.95	0.58	0.98	0.97
Kidney cortex	0.97	0.22	0.99	0.99

Conditional Posterior Probability of Colocalization, PP4/ (PP3 + PP4) the results of the colocalization analysis between each trait and *ACE* eQTL

### Locus discovery analysis results

We moved to a broad-scale locus discovery effort using bivariate GWAS to detect novel pleiotropic loci that were not previously associated with AD or the eleven cardiometabolic traits of interest (“Methods”). After applying a battery of filters to identify the subset of loci with positive evidence of pleiotropy and novelty, we were left with thirteen independent loci (Additional file 1 - Supplementary Table 3).

We next performed trait-trait colocalization analysis and found that three of the thirteen independent loci colocalized (Table 5). Thus, there was strong evidence of a shared causal SNP between AD and cardiometabolic traits at these loci. Among the thirteen independent loci was a locus with low-frequency exonic lead SNP with a bivariate *P* value of 7 × 10^− 8^. Due to the lead SNP being a low-frequency SNP, it had very little LD with other SNPs, which was not conducive to colocalization analyses (Table 5).

**Table 5:** Locus discovery analysis pleiotropic loci

Cardiometabolic trait	Locus name	Lead SNP	Chr	Position GRCh37	Effect allele/ other allele	Direction of effect AD/CM	Effect allele frequency	Bivariate *P* value	AD *P* value	Cardiometabolic trait *P* value	Conditional posterior probability of colocalization	Cardiometabolic GWAS
LDL	*DOC2A*	rs11642612	16	30030195	A/C	+/+	0.62	2.19e−08	5.32e−06	2.68e−06	0.98	Klarin et al. [24]
HDL	*SPPL2A*	rs12595082	15	51007729	C/T	+/+	0.81	3.27e−08	1.66e−06	1.09e−05	0.95	Klarin et al. [24]
BFP	*CCNT2*	rs10496731	2	135597628	G/T	+/−	0.48	1.74e−08	1.72e−05	2.67e−05	0.94	Lu et al. [23]
DBP	*DOCK4*	rs144867634	7	111580166	T/C	+/+	0.98	8.33e−08	5.36e−05	1.089e−07	NA	Evangelou et al. [20]

*Chr* chromosome of the SNP. Direction of effect first position is the direction of effect of the effect allele on AD and the cardiometabolic trait. Effect allele frequency from the Jansen et al. [17] allele frequency. Conditional posterior probability of colocalization, PP4/ (PP3 + PP4) the results of the trait-trait colocalization analysis

To identify candidate causal genes, we performed single-tissue-eQTL colocalization analysis at the three loci that were conducive to colocalization analysis. We found that all three loci colocalized with one or more single-tissue eQTL signals from GTEx v7 (Additional file 3 - Supplementary Table 12).

The first novel pleiotropic signal we detected was between LDL and AD at the *DOC2A* locus (Additional file 1 - Fig. S4). This region has been implicated in other cardiometabolic and neurological traits in previous single-trait GWAS [26]. The lead SNP, rs11642612, is in LD (1 kG EUR *r*^2^=0.753) with SNPs that are associated with BMI and schizophrenia [26]. Single-tissue eQTL colocalization found that this pleiotropic signal colocalized with several eQTL signals, but it most strongly colocalized with an eQTL for *DOC2A* in pancreatic tissue (Additional file 3 - Supplementary Table 12).

The next pleiotropic signal was between AD and HDL at the *SPPL2A* locus with the lead SNP rs12595082 (Fig. S5). This locus was reported as near genome-wide significantly associated with late-onset AD in Kunkle et al. [31]; however, our bivariate result is the first analysis to detect it at genome-wide significance. This locus was also detected in our AD and DBP bivariate GWAS with the lead SNP rs12440570. Colocalization analysis suggests that the AD, HDL, and DBP association peaks all colocalize with each other (conditional probability of colocalization = 0.81) (Additional file 1 - Supplemental Methods: MOLOC for the *SPPL2A* locus) [37]. The single-tissue eQTL analysis showed that this signal colocalized with eQTLs for multiple nearby genes (Additional file 3 - Supplementary Table 12).

We detected an opposite direction of effect pleiotropic signal between AD and BFP at the *CCNT2* locus (Additional file 1 – Figure S6). Several other neurological and cardiometabolic traits have been associated with this locus [26]. The lead SNP, rs10496731, is in LD with SNPs that are associated with Parkinson’s disease (1 kG EUR *r*^2^> 0.378), and DBP (1 kG EUR *r*^2^> 0.978) from single-trait GWAS [26]. Single-tissue-eQTL colocalization analysis indicated this signal colocalized with eQTLs for *CCNT2* in skin and *AC016725.4* in testis (Additional file 3 - Supplementary Table 12).

**Fig. 3:**
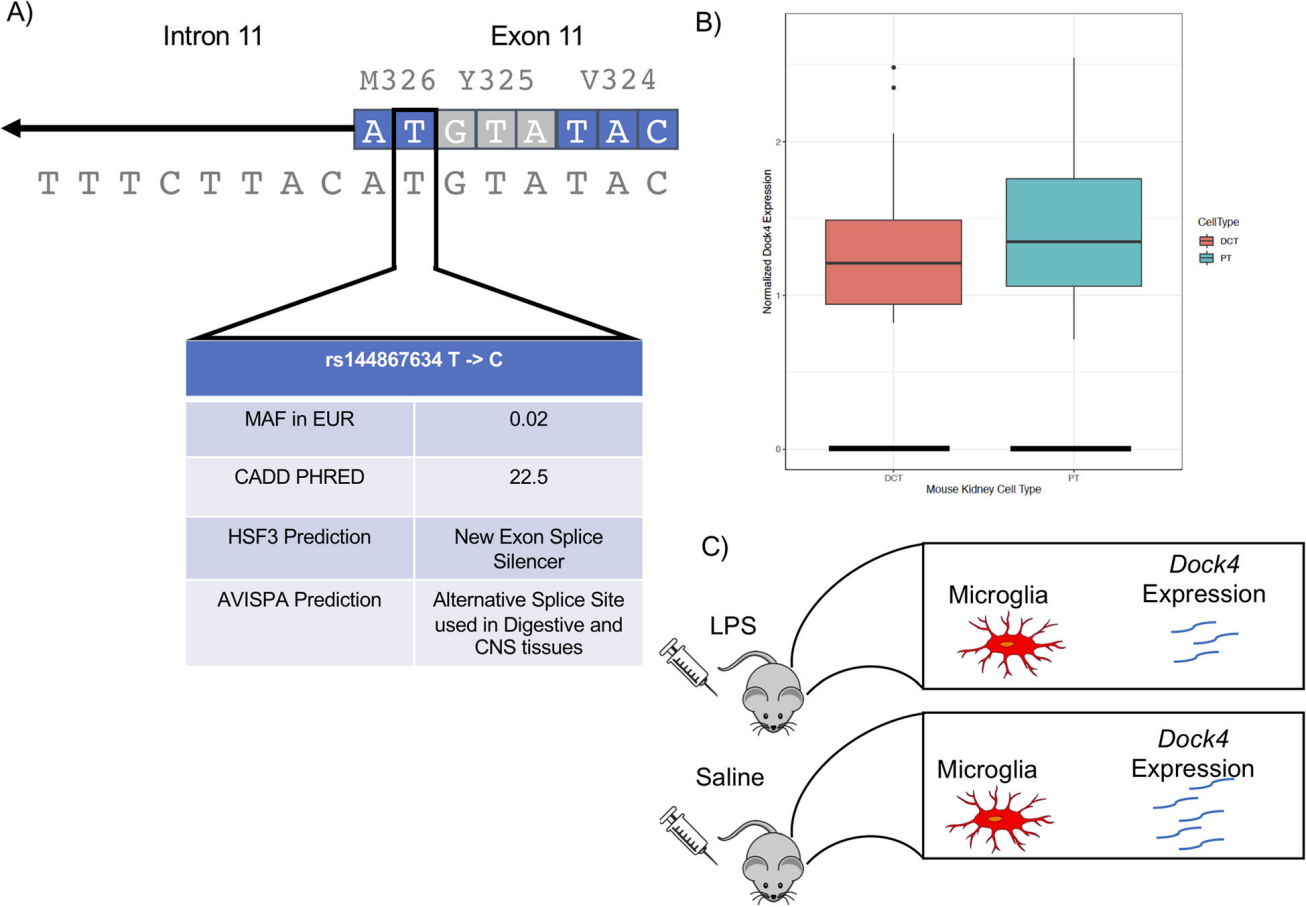
*DOCK4* locus. Evidence that supports rs144867634 being the causal variant for the pleiotropic signal at the *DOCK4* locus. **a** In silico evidence that rs144867634 alters *DOCK4* splicing. The variant is at the splice junction and is predicted to alter splicing by Human Splice Finder 3 (HSP3) and ASVIPA. **b** Single-cell mouse kidney data show that *Dock4* is expressed by proximal convoluted tubule cells (PT) (128 PT cells of 26,482 assayed have clear evidence of expressing *Dock4*) and distal convoluted tubule cells (DCT) (27 DCT cells of 8544 assayed have clear evidence of expressing *Dock4*) (Park et al. 2018). **c** Mouse brain single-cell data show that *Dock4* expression is reduced in microglia when mice have a neuroinflammatory response induced by endotoxin lipopolysaccharide (LPS) injections (Srinvasan et al. 2016)

The pleiotropic signal we detected at the *DOCK4* locus was between AD and DBP, with rs144867634 as the lead SNP (Fig. 3). rs144867634 is a low-frequency missense variant that is two bases away from the 3′ splice junction of the eleventh exon of *DOCK4* (Fig. 3a). This led us to evaluate whether rs144867634 alters the splicing of *DOCK4*. According to our in silico evaluation of rs144867634’s effect on splicing, it is likely that it alters the splicing of *DOCK4*, leading to exon 11 being spliced out of the *DOCK4* transcript (Fig. 3) (Additional file 1 - Supplemental Methods and Supplementary Tables 13–15).

## Discussion

Here, we demonstrate that a bivariate GWAS method coupled with colocalization analysis enabled the detection of pleiotropic loci between these complex traits and identification of plausible causal genes and potential therapeutic targets. We detected three AD-associated loci with previously unknown pleiotropy for cardiometabolic traits and four loci that were pleiotropic and novel for both AD and the pertinent cardiometabolic trait, all of which we were able to map to one or more candidate causal genes. While our manuscript was under consideration, we note that a report was posted which indicated the *DOC2A* locus is a genome-wide significant AD locus supporting our results [38].

Our findings support those of previous pleiotropy studies between these traits: that there is a complex genetic relationship between AD and cardiometabolic traits involving both vertical and horizontal pleiotropy [4]. Many of the loci suggest a mechanism where AD and cardiometabolic traits have different causal tissues for the two traits. Further evaluation of the loci we reported could aid in predicting the side effects of medications and for drug repurposing for AD and cardiometabolic diseases.

The candidate causal genes we identified through single-tissue-eQTL colocalization analysis support the roles of blood pressure and immune response in both AD and cardiometabolic traits. Three of the pleiotropic loci we report implicate blood pressure mechanisms involved in the pleiotropic relationship at the locus, and four loci had candidate causal genes that have been shown to be involved in immune responses. While these mechanisms make sense given that hypertension and inflammation have both been linked with AD and cardiometabolic diseases, they have not been prevalent in the discussion of pleiotropy between these traits [4, 34, 39–42]. We did not perform experiments to identify causal variants at these loci in this study. However, for some of these loci, previous work has identified plausible causal variants tagged by the pleiotropic signal (e.g., the ALU insertion/deletion at the *ACE* locus) [32].

The pleiotropic signal at the *ACE* locus allowed us to shed more light on a locus that is potentially clinically relevant, but complex. *ACE* is an important enzyme in the renin-angiotensin system, and it is the target gene of ACE inhibitors, a common hypertension medication. This locus has also been well studied from an AD perspective [32, 33, 35]. We found that the allele associated with increased risk of AD and decreased DBP and SBP was associated with decreased *ACE* expression in brain tissues and most other tissues, but increased *ACE* expression in transverse colon and kidney (Fig. 2b and Additional file 1 - Supplemental Table 11). These opposite direction of effect single-tissue *ACE* eQTLs appear to colocalize with one another and be independent of a lung *ACE* eQTL nearby (Table 3 and Table 4). However, we cannot exclude the possibility of two causal variants that are both in LD (1 kG EUR *r*^2^ > 0.8) with the lead SNP of the pleiotropic signal, rs4308.

The decrease in blood pressure could be due to the increase in *ACE* expression in the kidney and the negative feedback loop between angiotensin II and renin (Fig. 2b,c) [43]. Our hypothesis is that increased expression of *ACE* in the kidney leads to increased levels of angiotensin II in the kidney. These locally increased levels of angiotensin II lead to reduced expression of renin, slowing the entire renin-angiotensin system, and decreasing blood pressure (Fig. 2c).

In recent years, the relationship between ACE inhibitors and AD has been an active field of study and has resulted in two leading hypotheses of how ACE inhibitors may alter AD risk [34, 39]. Several studies have found that patients on ACE inhibitors that cross the blood-brain barrier (centrally acting) are at reduced risk of dementia and have improved cognitive ability. Other studies have found evidence that patients taking ACE inhibitors have decreased cognitive function and increased levels of β-amyloid (Aβ) protein in their central nervous system; these results were also replicated in mice [40]. This is thought to be due to ACE’s ability to cleave Aβ42 to Aβ40, which is a form of Aβ that is less pathogenic than Aβ42 due to it being less prone to aggregate in the brain [40]. Increases in Aβ42 to Aβ40 ratios have been associated with the *PSEN1* and *PSEN2* mutations in the familial form of AD [44]. Our results support this second hypothesis, that reduced ACE activity in the brain leads to more Aβ42, which in turn could lead to more Aβ plaques and an increase in AD risk (Fig. 2b). Our findings suggest that further work should be done to evaluate the role of ACE therapeutics for risk of AD.

The BFP and AD pleiotropic signal at the *CCNT2* locus has a particularly compelling potential mechanism. Single-tissue-eQTL colocalization analysis detected colocalization between the bivariate signal and an eQTL for *CCNT2* in skin tissue (Table 5 and Additional file 1 – Figure S6). The gene *CCNT2* is a strong candidate for being involved with both the BFP and the AD association. *CCNT2* has been shown to be important in adipose biology [45]. Human *CCNT2* knockout adipocytes have altered adipogenesis gene expression and decreased secretion of the hunger inhibiting hormone leptin, which is consistent with increased BFP [45]. CCNT2 has also been shown to be used by herpes simplex virus 1 (HSV-1) when it transcribes its genome [46]. This is a plausible link to AD due to the hypothesis that HSV-1 can trigger amyloid plaques [47, 48].

Finally, our results suggest that *DOCK4* is the putative causal gene for the pleiotropic signal between DBP and AD at the *DOCK4* locus, since the lead SNP is a low-frequency exonic variant in *DOCK4* that is predicted to lead to exon 11 of *DOCK4* being spliced out of the *DOCK4* transcript (Fig. 3a). For these reasons, and the fact that the rare allele is associated with lower risk of AD and reduced DBP, *DOCK4* is our strongest candidate for a novel therapeutic target. The human genetics data observed here is consistent with the simple hypothesis that reduced efficacy of *DOCK4* in vivo could treat both hypertension and AD. There is already evidence that *DOCK4* could be involved with AD and DBP. Previous genetic studies have shown that *DOCK4* variants are associated with multiple neurological phenotypes, and *DOCK2*, the other member of *DOCK4*’s protein subfamily, expression is increased in the microglia of patient’s with AD [49, 50]. It has also been shown that *Dock4* expression in mouse microglia is altered when mice are given an endotoxin lipopolysaccharide (LPS) injection to induce a neuroinflammatory response (Fig. 3c) [51]. *DOCK4* could also affect DBP through changes in kidney function. *DOCK4* is expressed in kidney in GTEx v8, and *Dock4* is expressed in mouse kidney proximal tubule cells and distal convoluted tubule cells. These cells are responsible for reabsorption of salts, sugars, and amino acids in the nephron of the kidney, and thus altering their function could change blood volume (Fig. 3b) [52, 53].

### Limitations

There are several limitations of our study. The Jansen et al. [17] AD GWAS and many of the cardiometabolic trait GWAS we used included individuals from the UK Biobank dataset. This sample overlap will increase the estimated covariance between our traits making the resulting bivariate *P* value more conservative for a locus that has the same direction of effect as the phenotypic correlation and less conservative when a locus has an opposite direction of effect. The overlapping samples may also inflate our posterior probability of colocalization between these traits. A phenotypic limitation of our study is that it is difficult to differentiate between AD, vascular dementia, and mixed dementia [54]. It is possible that some of the pleiotropic loci we detected are due to vascular or mixed dementia patients being included in this AD cohorts, particular since Jansen et al. include some Proxy-AD patients [17] .

## Conclusion

We have shown that bivariate GWAS paired with colocalization analysis can be an effective way to detect pleiotropic loci between complex traits and generate hypotheses as to why these loci are pleiotropic. We detected seven loci that have evidence of being pleiotropic between AD and a cardiometabolic trait, and we were able to identify candidate causal genes for all of these loci. Two loci seem to stand out in their potential to improve our ability to prevent and treat AD. The first is the *ACE* locus, which provides more evidence to support a potential link between AD risk and ACE inhibitors. The other is the *DOCK4* locus which is our most promising candidate for a novel therapeutic target. Our results may aid in resolving the etiology of AD and help identify new therapeutic targets for this disease. AD is a complex disease, and we expect that applying this method to other traits that have been associated with AD, such as educational attainment and immune traits, should also lead to novel pleiotropic loci, new candidate causal genes, and a better understanding of AD [42, 55, 56].

## Supplementary Information

**Additional file 1: **Supplemental Methods – Details on how we collected the GWAS summary statistics and how the statistical analyses were performed. Figure S1. *ADAM10* locus – LocusZoom plots of the pleiotropic signals and the *MINDY2* eQTL signal at the *ADAM10* locus. Figure S2. *ADAMTS4* locus – LocusZoom plots of the pleiotropic signals and *NDUFS2* eQTL signal at the *ADAMTS4* locus. Figure S3. T2D at *ACE* locus – LocusZoom plots of the T2D signals at the *ACE* locus. Figure S4. *DOC2A* locus – LocusZoom plots of the pleiotropic signals and *FAM57B a*nd *DOC2A* eQTL signal at the *DOC2A* locus. Figure S5. *SPPL2A* locus – LocusZoom plots of the pleiotropic signals and *SPPL2A* eQTL signal at the *SPPL2A* locus. Figure S6. *CCNT2* locus – LocusZoom plots of the pleiotropic signals and *CCNT2* eQTL signal at the *CCNT2* locus. Supplementary Table 1. Bivariate normal estimates for the SNP Z-scores of each bivariate GWAS. Supplementary Table 2. Number of SNPs that passed each filter for the AD-centric analysis. Supplementary Table 3. Number of SNPs that passed each filter for the locus discovery analysis. Supplementary Table 4. Single-tissue eQTLs for rs4308 downloaded from GTExPortal. Supplementary Table 5. Single-tissue eQTLs for rs442495 downloaded from GTExPortal. Supplementary Table 6. Single-tissue eQTLs for rs4575098 downloaded from GTExPortal. Supplementary Table 7. Single-tissue eQTLs for rs10496731 downloaded from GTExPortal. Supplementary Table 9. Single-tissue eQTLs for rs12595082 downloaded from GTExPortal. Supplementary Table 10. List of approximate conditional analyses. Supplementary Table 11. AD-centric bivariate analysis single-tissue-eQTL results. Supplemental Table 13. Human Splice Finder 3 results for rs144867634. Supplemental Table 14. AVISPA results on rs144867634 A allele. Supplemental Table 15. AVISPA results on rs144867634 G allele.

**Additional file 2:.** Supplemental Table 8. Single-tissue eQTLs for rs11642612 downloaded from GTExPortal.

**Additional file 3:.** Supplemental Table 12. Locus discovery analysis single-tissue-eQTL results.

## Data Availability

The software supporting the conclusions of this article are available here: All code is available under a GNU Public License v3 license: Language: R (requires version 3.5 or later). The bivariate GWAS code generated during this study are available at AD_and_Cardiometabolic_Trait_Bivariate_Scans https://github.com/wpbone06/AD_and_Cardiometabolic_Trait_Bivariate_Scans. The eQTL colocalization code generated during this study are available at GTEx_v7_eQTL_colocalizer https://github.com/wpbone06/GTEx_v7_eQTL_colocalizer. The datasets supporting the conclusions of this article are available here: GWAS summary statistics data used in the paper are available at: AD data PMID:30617256 https://ctg.cncr.nl/software/summary_statistics, BFP data PMID:26833246 https://walker05.u.hpc.mssm.edu/, BMI data PMID: 30124842 https://portals.broadinstitute.org/collaboration/giant/index.php/GIANT_consortium_data_files#2018_GIANT_and_UK_BioBank_Meta_Analysis_for_Public_Release, CHD data PMID: 29212778 www.cardiomics.net, DBP data PMID: 30224653, HDL data PMID: 30275531, LDL data PMID: 30275531, SBP data PMID: 30224653, TC data PMID: 30275531, TG data PMID: 30275531, T2D data PMID: 30297969 http://diagram-consortium.org/downloads.html, WHRadjBMI data PMID: 30239722 https://zenodo.org/record/1251813#.XOxslYhKg2w Access to the MVP lipids data can be obtained from dbGAP (phs001672.v4.p1, pha004828.1, pha004831.1, pha004837.1, pha004834.1) and GLGC European ancestry only data can be obtained at: http://csg.sph.umich.edu/willer/public/lipids/ or http://lipidgenetics.org/. The GTEx eQTL data used in these analyses are available at: https://www.gtexportal.org/ and https://www.gtexportal.org/home/datasets .

## Abbreviations

AD: Alzheimer’s disease
BMI: Body mass index
BFP: Body fat percentage
CHD: Coronary heart disease
DBP: Diastolic blood pressure
eQTL: Expression quantitative trait loci
GTEx: Genotype-Tissue Expression
GWAS: Genome-wide association study
HDL: High-density lipoproteins
LDL: Low-density lipoproteins
SBP: Systolic blood pressure
SNP: Single-nucleotide polymorphism
T2D: Type II diabetes
TC: Total cholesterol
TG: Triglycerides
WHRadjBMI: Waist-hip ratio adjusted for BMI
