# Supplementary material for: Multi-trait association studies discover pleiotropic loci between Alzheimer’s disease and cardiometabolic traits
Source: Alzheimers Res Ther. 2021 Feb 4;13:34. doi: 10.1186/s13195-021-00773-z (PMC7860582; doi:10.1186/s13195-021-00773-z)
Supplement: Supplementary file 1 — Additional file 1: Supplemental Methods – Details on how we collected the GWAS summary statistics and how the statistical analyses were performed. Figure S1. ADAM10 locus – LocusZoom plots of the pleiotropic signals and the MINDY2 eQTL signal at the ADAM10 locus. Figure S2. ADAMTS4 locus – LocusZoom plots of the pleiotropic signals and NDUFS2 eQTL signal at the ADAMTS4 locus. Figure S3. T2D at ACE locus – LocusZoom plots of the T2D signals at the ACE locus. Figure S4. DOC2A locus – LocusZoom plots of the pleiotropic signals and FAM57B and DOC2A eQTL signal at the DOC2A locus. Figure S5. SPPL2A locus – LocusZoom plots of the pleiotropic signals and SPPL2A eQTL signal at the SPPL2A locus. Figure S6. CCNT2 locus – LocusZoom plots of the pleiotropic signals and CCNT2 eQTL signal at the CCNT2 locus. Supplementary Table 1. Bivariate normal estimates for the SNP Z-scores of each bivariate GWAS. Supplementary Table 2. Number of SNPs that passed each filter for the AD-centric analysis. Supplementary Table 3. Number of SNPs that passed each filter for the locus discovery analysis. Supplementary Table 4. Single-tissue eQTLs for rs4308 downloaded from GTExPortal. Supplementary Table 5. Single-tissue eQTLs for rs442495 downloaded from GTExPortal. Supplementary Table 6. Single-tissue eQTLs for rs4575098 downloaded from GTExPortal. Supplementary Table 7. Single-tissue eQTLs for rs10496731 downloaded from GTExPortal. Supplementary Table 9. Single-tissue eQTLs for rs12595082 downloaded from GTExPortal. Supplementary Table 10. List of approximate conditional analyses. Supplementary Table 11. AD-centric bivariate analysis single-tissue-eQTL results. Supplemental Table 13. Human Splice Finder 3 results for rs144867634. Supplemental Table 14. AVISPA results on rs144867634 A allele. Supplemental Table 15. AVISPA results on rs144867634 G allele. [file 13195_2021_773_MOESM1_ESM.pdf]

Supplemental Methods:

Web resources:

GWAS Catalog, https://www.ebi.ac.uk/gwas/docs/file-downloads

LocusZoom, http://locuszoom.org/genform.php

LDlink, https://ldlink.nci.nih.gov/?tab=ldassoc

MyVariant.info, https://myvariant.info/

Combined Annotation Dependent Depletion (CADD), https://cadd.gs.washington.edu/score

Human Splice Finder 3, http://www.umd.be/HSF3

GWAS data collection:

AD:

We downloaded the summary statistics from https://ctg.cncr.nl/software/summary_statistics (01/22/2019).

BFP:

We downloaded the summary statistics from https://walker05.u.hpc.mssm.edu/ (03/30/2018). GRCh37 positions were added by merging these data on rsIDs with the van der Harst et al. 2018 data.

BMI:

We downloaded the summary statistics from https://portals.broadinstitute.org/collaboration/giant/index.php/GIANT_consortium_data_files#2018_GIANT_and_UK_BioBank_Meta_Analysis_for_Public_Release (10/29/2018).

CHD:

We downloaded the van der Harst et al. 2018 data from www.cardiomics.net

(02/13/2018).

Blood pressure data (DBP and SBP):

We received these data from a corresponding author of Evangelou et al. 2018 (12/07/2018). We added rsIDs to the file by merging these data on GRCh37 positions based unique IDs with the full list of SNPs from dbSNP v150 data.

Lipids data (HDL, LDL, TC, and TG):

We used the European ancestry data from the Klarin et al. 2018 data. We requested the European ancestry lipid data from the Million Veterans Program (01/07/2018) and meta-analyzed these data with the Willer et al. 2013 data using *PLINK*(1). We then removed SNPs from the meta-analysis file that had an imputation score less than 0.8.

Access to the MVP lipids data can be obtained from dbGAP (phs001672.v4.p1, pha004828.1, pha004831.1, pha004837.1, pha004834.1) and GLGC European ancestry only data can be obtained at: http://csg.sph.umich.edu/willer/public/lipids/ or http://lipidgenetics.org/.

T2D:

We downloaded these data from http://diagram-consortium.org/downloads.html (01/04/2019). We added rsIDs by merging these data on GRCh37 positions based unique IDs with full list of SNPs from the Jansen et al. 2019 AD data file.

WHRadjBMI:

We downloaded these data from https://zenodo.org/record/1251813#.XOxslYhKg2w (5/28/2019).

Bivariate GWAS:

The bivariate GWAS method uses GWAS summary statistics from two traits to perform a statistical test of whether each SNP is associated with one or both of the traits from the GWAS data. The method does this by converting the P-value, effect estimates, and standard errors from the summary statistics files to Z-scores(2–4). Given that Z-scores should be standard normal distributed, we can estimate the bivariate normal distribution of the SNP Z-scores. We can then perform an association test for each SNP using a chi-squared test with two degrees of freedom to calculate a bivariate P-value(2–4).

The first step to perform a bivariate scan is to harmonize the alleles of the summary statistics data from the two GWAS studies using the harmonise_data function in *MRbase*(5). We next use *PLINK* to obtain independent SNPs using the command "--indep-pairwise 1000 5 0.2" with the 1000 Genomes phase 3 European ancestry (1kG EUR) as the linkage reference panel(6). We estimate the mean and covariance matrix of the bivariate normal distribution of SNP Z-scores using the independent SNPs. As described above, we then used these parameters to calculate a bivariate P-value for each harmonized SNP(2–4). Independent associated loci were produced using the "--clump-r2 0.2" command in *PLINK*(1). We further filtered independent associated loci differently for the "AD-centric" and the "locus discovery" experiment to detect loci of interest (see Materials and Methods: AD-centric Analysis and Locus Discovery Analysis).

MOLOC for the *SPPL2A* locus:

To perform a colocalization analysis between the AD, HDL, and DBP association peaks at the *SPPL2A* locus we used the *MOLOC* library in R(7). *MOLOC* is an extension of the *COLOC*

software that calculates the posterior probability of colocalization between 2 or more traits. We ran *MOLOC* on the same input data that we used to run *COLOC* between the AD and HDL association peaks and the AD and DBP association peaks. We then calculated the conditional probability of colocalization as the posterior probability of colocalization conditioned on the presence of a signal for each trait.

***In silico* variant assessment of rs144867634:**

We searched the MyVariant database and the Combined Annotation Dependent Depletion (CADD) data base to review the functional annotation of rs144867634 (http://myvariant.info/v1/variant/chr7:g.111580166T%3EC and https://cadd.gs.washington.edu/snv/GRCh37-v1.4/7:111580166_T_C)(8,9). The collection of these annotations agreed that this variant was a missense variant in *DOCK4*. However, the predicted change in amino acid from methionine to valine was not likely to be deleterious to the *Dock4* protein function. Thus, we wanted to determine what the predicted effect of rs144867634 would be on the splicing of *DOCK4* via Human Splice Finder 3 (HSF3) and AVISPA(10,11).

Human Splice Finder analysis:

Human Splice Finder 3 (HSF3) is an ensemble method intended to predict the effect of exonic and intronic variants on the splicing of a gene(10). We used the analyze mutations function of HSF3, and provided the cDNA position of rs144867634 (c.976A>G) in the *DOCK4* transcript ENST00000428084 on February 28, 2019. Results can be seen in Supplemental Table 12. The results suggest that the C allele of rs144867634 will lead to a new exonic splice silencer (ESS) and will alter an exonic splice enhancer (ESE).

AVISPA

AVISPA is a web-tool for splicing prediction and analysis of cassette splicing events. Using splicing features derived from exonic and intronic regions of the exon triplet, AVISPA predicts if the central exon is alternatively or constitutively spliced and displays tissue-specific splicing variations in inclusion levels of the central exon in 4 tissue groups: central nervous system, muscle, digestive and embryonic tissue group. We ran AVISPA on the target exon (GRCh37 chr7:111580165-111580297) containing rs144867634, along with two flanking exons (GRCh37 chr7:111584866-111584926, chr7:111575595-111575683) (Supplemental Table 15 and Supplemental Table 16). Two configurations of input genomic sequences were provided to AVISPA, one for each allele of rs144867634. For both of these configurations, AVISPA predicted if the exon is alternative or constitutive and tissue-specific splicing variations (differential inclusion or exclusion of the central exon) in each of the 4 tissues groups. For each of these predictions, we report false positive rate, rank and sensitivity of the prediction. The results suggest that exon 11 is not a constitutively included exon and that the low frequency G allele alters splicing of exon 11 of *DOCK4* in digestive and central nervous system tissues.

Figure S1. ***ADAM10*** **locus**

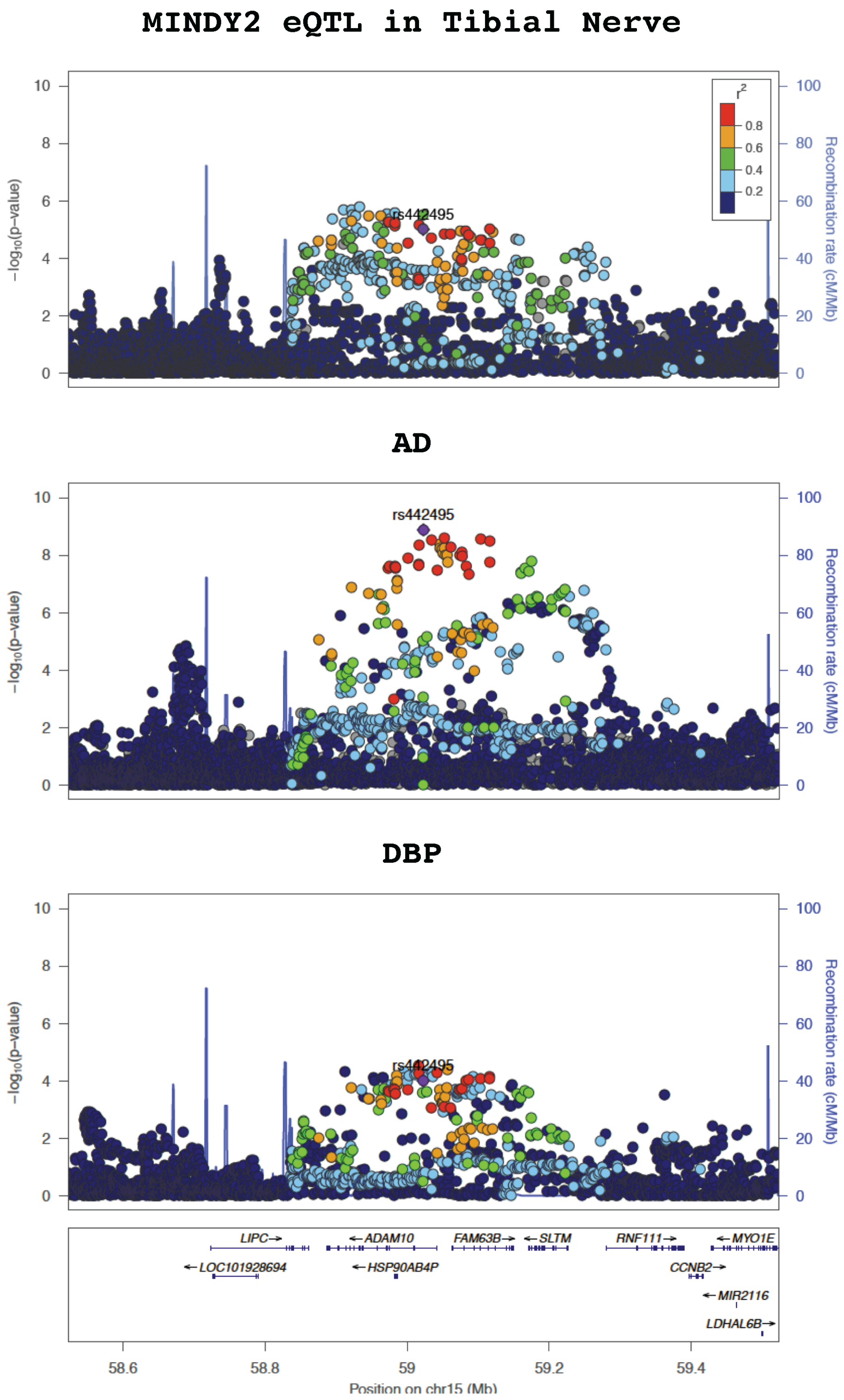

Pleiotropic signal between DBP and AD and the *MINDY2* eQTL signal at the *ADAM10* locus. The top panel is the eQTL signal for the gene *MINDY2* in tibial nerve from GTEx v7. The middle panel show the AD association peak at this locus. The bottom panel shows the association peak for BFP at this locus.

Figure S2. ***ADAMTS4*** **locus**

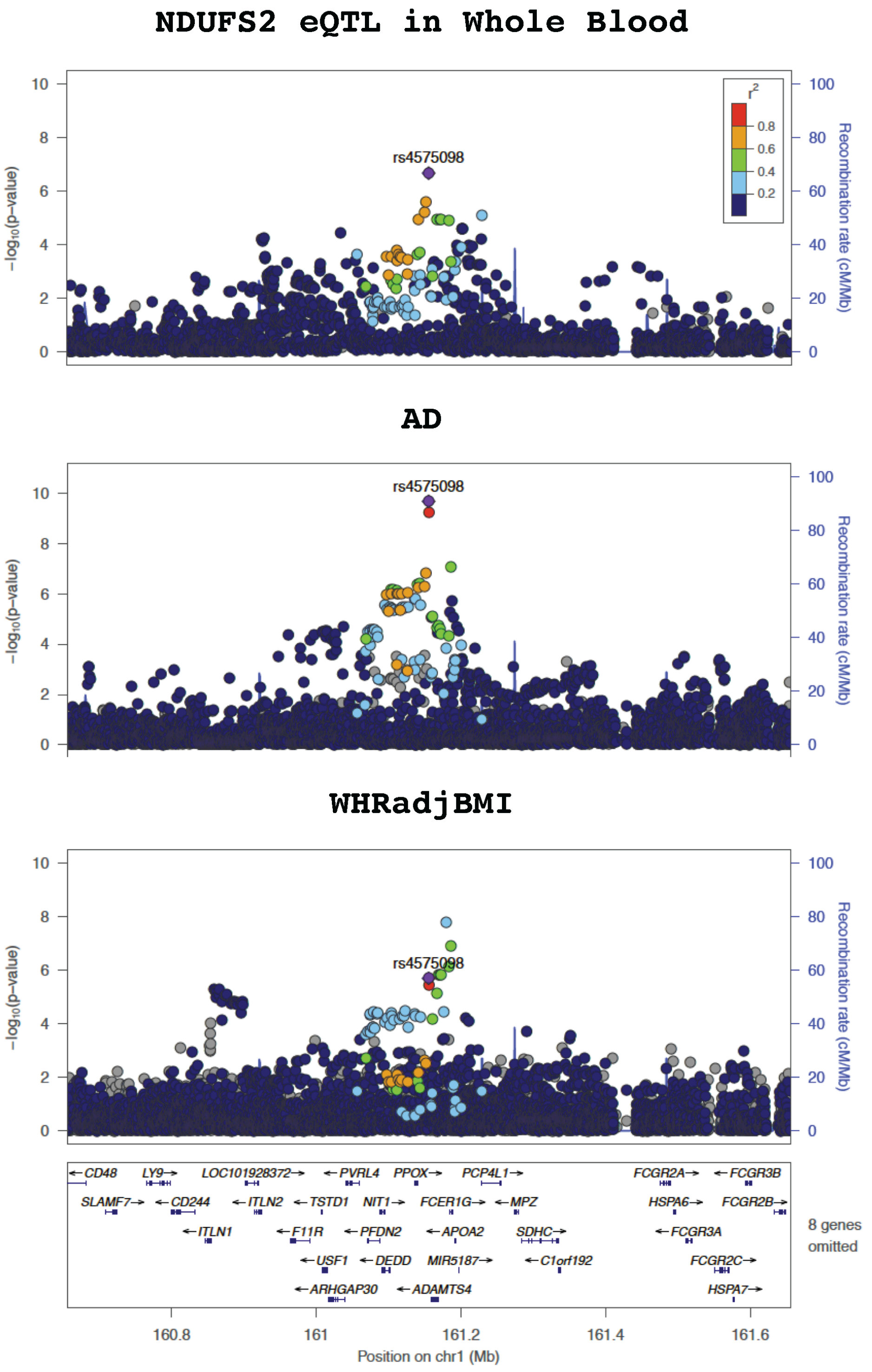

Pleiotropic signal between WHRadjBMI and AD and the *NDUFS2* eQTL signal at the *ADAMTS4* locus. The top panel is the eQTL data for the gene *NDUFS2* in whole blood from GTEx v7. The

middle panel show the AD association peak at this locus. The bottom panel shows the association peak for BFP at this locus.

Figure S3. T2D at *ACE* locus

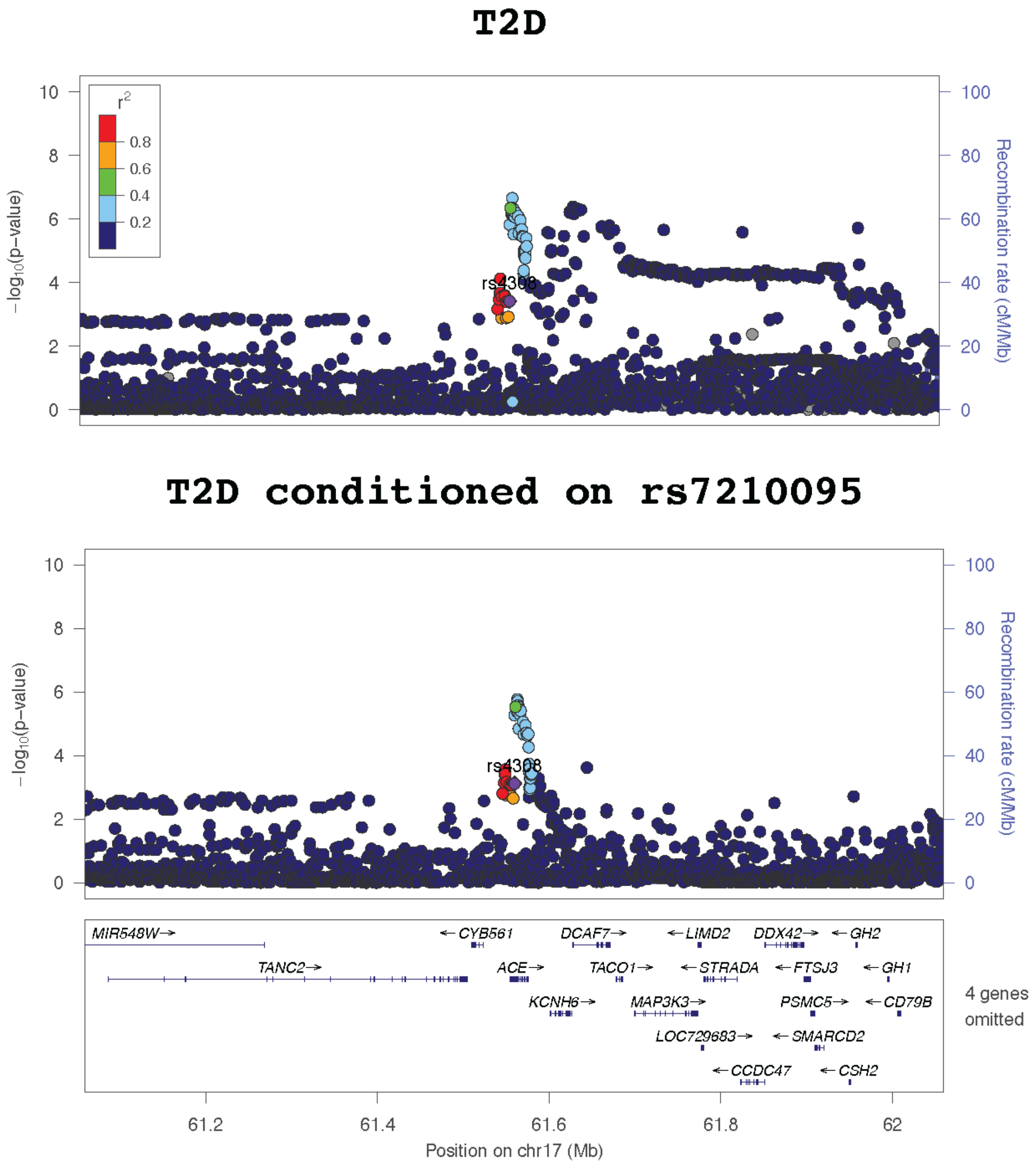

T2D signal at the *ACE* locus. The top panel is the T2D association peak at this locus. The bottom panel shows the same association peak after conditioning on rs7210095.

Figure S4. *DOC2A* Locus

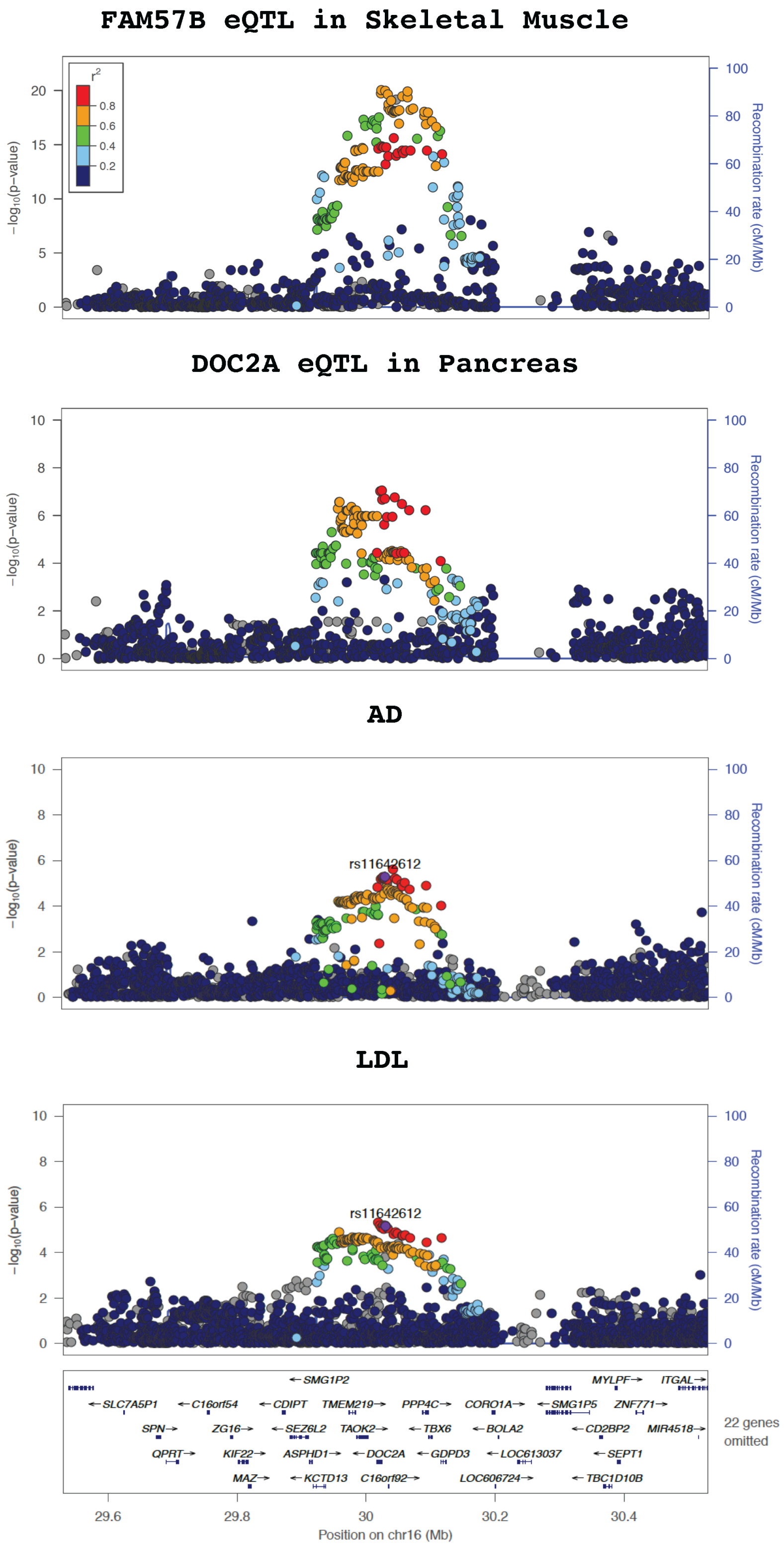

Pleiotropic signal between LDL and AD and the *FAM57B* and *DOC2A* eQTL signals at the *DOC2A* locus. The top two panels are the eQTL data for the gene FAM57B in skeletal muscle and *DOC2A* in pancreas from GTEx v7. The second from bottom panel show the AD association peak at this locus. The bottom panel shows the association peak for LDL at this locus.

Figure S5. ***SPPL2A*** **Locus**

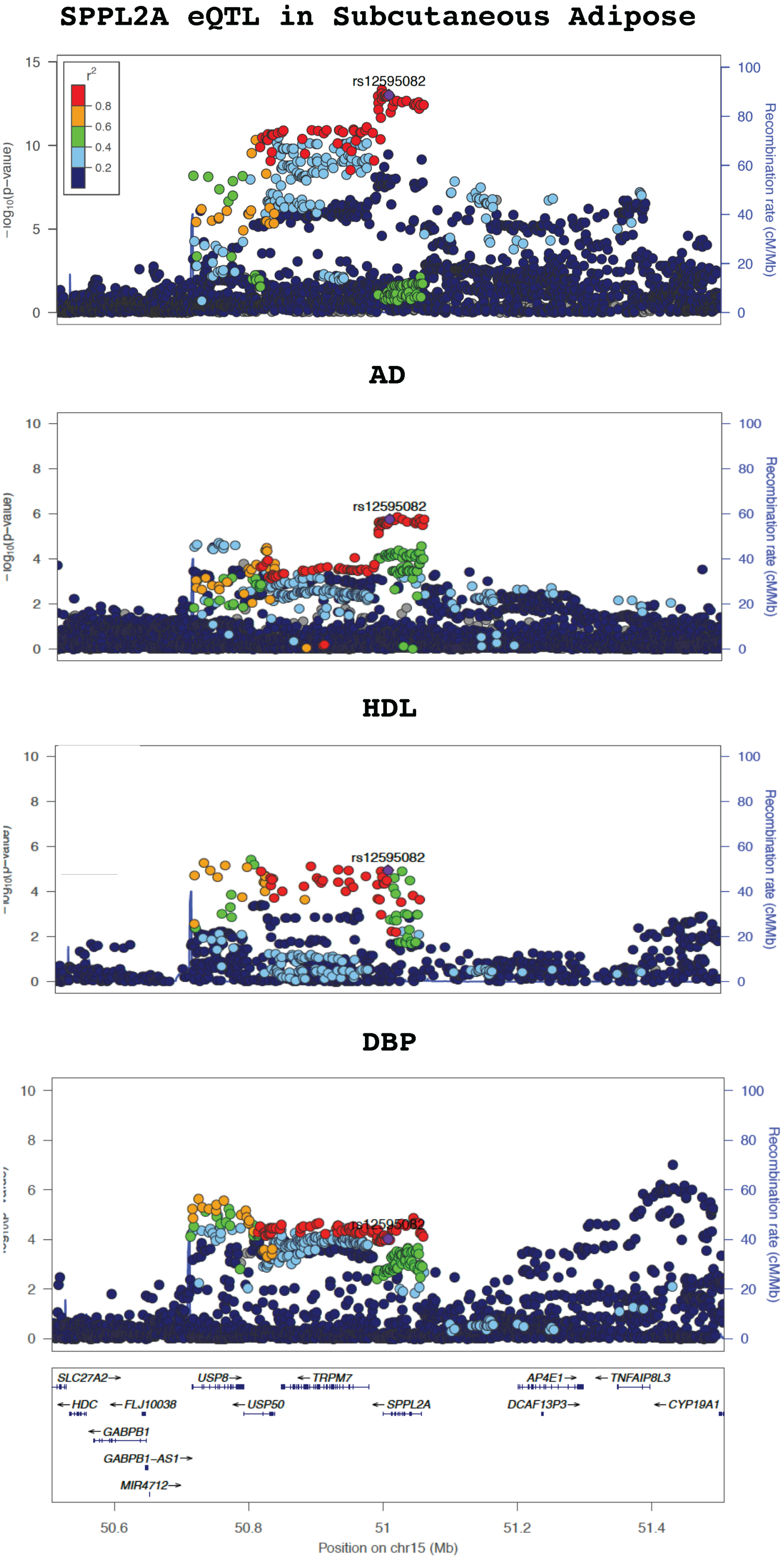

Pleiotropic signal between HDL, DBP, and AD the *SPPL2A* eQTL signal at the *SPPL2A* locus. The top panel is the eQTL data for the gene *SPPL2A* in subcutaneous adipose tissue from GTEx v7. The second panel shows the AD association peak at this locus. The bottom two panels show the association peaks for HDL and DBP at this locus.

Figure S6. ***CCNT2*** **Locus**

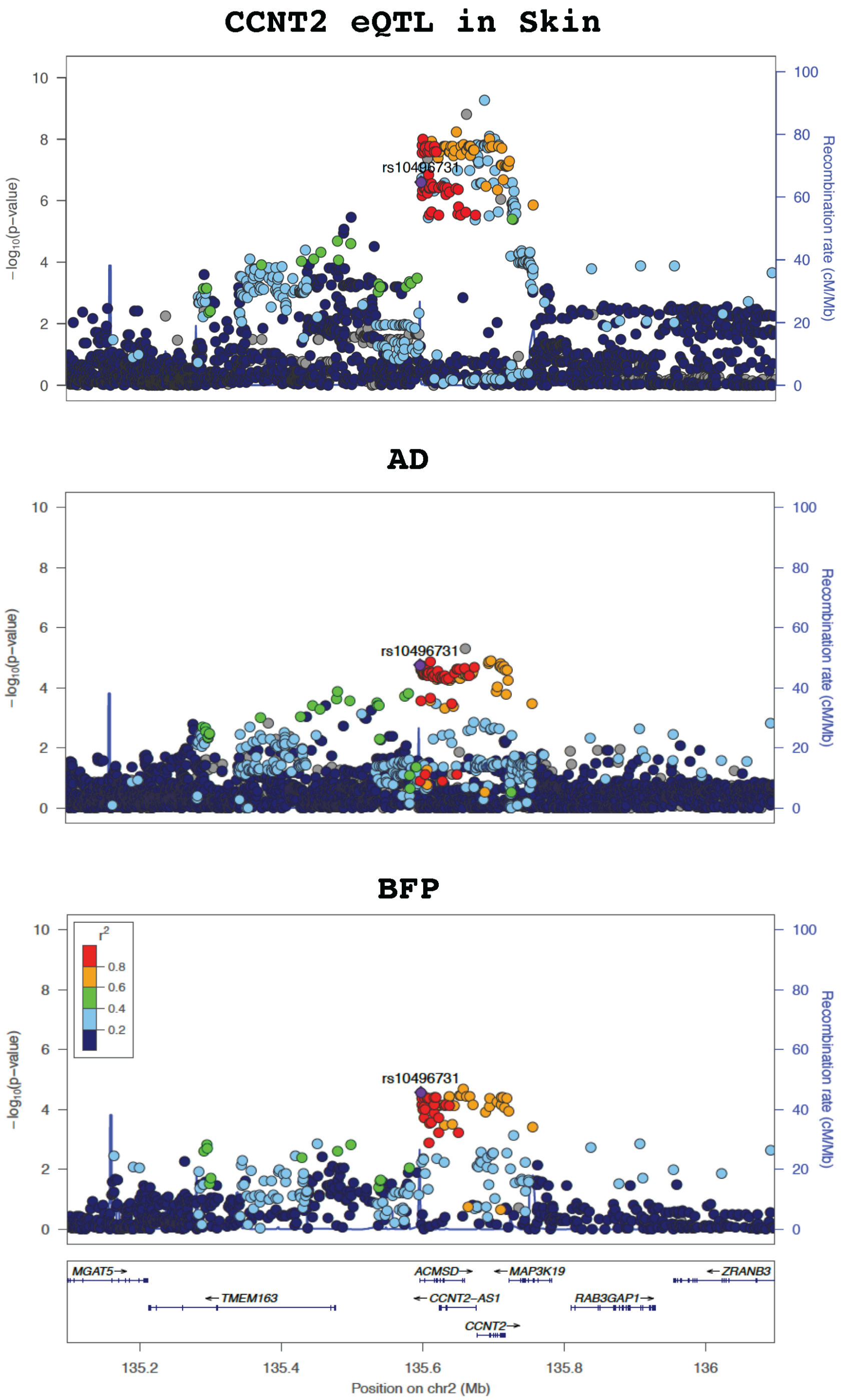

Pleiotropic signal between BFP and AD and the *CCNT2* eQTL signal at the *CCNT2* locus. The top panel is the eQTL data for the gene *CCNT2* in skin tissue from GTEx v7. The middle panel

show the AD association peak at this locus. The bottom panel shows the association peak for BFP at this locus.

Supplementary Table 1. Bivariate normal estimates for the SNP Z-scores of each bivariate GWAS

Trait	AD estimate of mean of Z scores	CM trait estimate of mean of Z scores	AD variance of Z scores	CM variance of Z scores	Covariance of Z scores
BFP	0.019069449	0.004121425	1.0478863729	0.9702646909	0.0009233505
BMI	0.02074968	0.01606504	1.05495557	2.34574289	0.02398266
CHD	0.01487529	0.01435294	1.03299962	1.13093119	0.01132046
DBP	0.022262691	-0.00498686	1.03489018	1.67147079	0.00735901
HDL	0.021838610	0.006383637	1.06941806	1.56940615	-0.01795169
LDL	0.021876493	-0.001275281	1.06665635	1.35407634	0.02037287
SBP	0.023884462	-0.009548027	1.037958337	1.685590553	0.009512961
TC	0.023248753	-0.003845626	1.06677737	1.42491297	0.02135301
TG	0.021774919	-0.003854562	1.0679191	1.5151773	0.0241633
T2D	0.01014445	0.19602156	1.02348921	1.16633941	0.01110318
WHRadjBMI	0.009765370	-0.002654256	1.023994769	1.268734246	0.006502855

Supplementary Table 2. Number of SNPs that passed each filter for the AD-centric analysis

Trait	Total Number of SNPs	Independent bivariate significant SNPs	AD associated SNPs that are possibly pleiotropic	Loci remaining after *HLA* and *APOE* loci filter	Colocalizing SNP
BFP	2,601,279	65	6	2	0
BMI	2,324,672	145	16	6	0
CHD	7,783,726	201	35	4	0
DBP	7,094,064	810	18	9	2
HDL	2,1784,81	413	26	0	0
LDL	2,174,511	380	35	0	0
SBP	7,023,719	732	22	5	1
T2D	11,215,941	528	21	4	0
TC	2,034,485	435	31	0	0
TG	2,174,226	401	24	3	0
WHRadjBMI	11,075,870	1,052	21	6	1
Total	55,502,493	5,162	255	39	4

Note: One of the novel loci was detected in both the SBP and AD and the DBP and AD-centric analyses.

Supplementary Table 3. Number of SNPs that passed each filter for the locus discovery analysis

Trait	Total Number of SNPs	Independent bivariate significant SNPs	Novel SNPs that are possibly pleiotropic	Colocalizing SNP
BFP	2,601,279	73	1	1
BMI	2,324,672	145	1	0
CHD	7,783,726	381	2	0
DBP	7,094,064	810	2	1
HDL	2,1784,81	413	2	1
LDL	2,174,511	380	1	0
SBP	7,023,719	732	0	1
T2D	11,215,941	528	2	0
TC	2,034,485	435	0	0
TG	2,174,226	401	0	0
WHRadjBMI	11,075,870	1,052	3	0
Total	55,502,493	5,350	14	4

Note: One of the novel loci was detected in both the HDL and AD and the DBP and AD bivariate GWAS.

Supplementary Table 4. Single-tissue eQTLs for rs4308 downloaded from GTExPortal Jan. 7th 2020

Gencode ID	Gene Symbol	P-Value	Normalized Effect Size	Tissue
ENSG00000159640.15	ACE	1.20E-27	-0.29	Cells - Cultured fibroblasts
ENSG00000159640.15	ACE	1.50E-22	0.46	Colon - Transverse
ENSG00000159640.15	ACE	4.20E-16	0.26	Lung
ENSG00000159640.15	ACE	2.90E-15	-0.18	Skin - Sun Exposed (Lower leg)
ENSG00000159640.15	ACE	2.70E-11	-0.15	Adipose - Subcutaneous
ENSG00000159640.15	ACE	1.80E-09	-0.41	Brain - Cerebellum
ENSG00000159640.15	ACE	2.20E-08	-0.18	Nerve - Tibial
ENSG00000159640.15	ACE	4.20E-08	0.56	Kidney - Cortex
ENSG00000159640.15	ACE	1.10E-07	-0.28	Adrenal Gland
ENSG00000159640.15	ACE	2.90E-07	-0.14	Skin - Not Sun Exposed (Suprapubic)
ENSG00000159640.15	ACE	3.30E-07	-0.38	Brain - Frontal Cortex (BA9)
ENSG00000159640.15	ACE	6.70E-07	-0.31	Brain - Cortex
ENSG00000159640.15	ACE	7.30E-07	-0.16	Adipose - Visceral (Omentum)
ENSG00000159640.15	ACE	9.20E-07	-0.39	Brain - Cerebellar Hemisphere
ENSG00000159640.15	ACE	0.0000019	-0.17	Artery - Aorta
ENSG00000159640.15	ACE	0.000019	-0.38	Brain - Anterior cingulate cortex (BA24)
ENSG00000224353.2	ACE3P	0.000024	0.14	Testis
ENSG00000213218.10	CSH2	0.0000013	-0.34	Testis
ENSG00000136485.14	DCAF7	1.90E-11	0.17	Lung
ENSG00000108592.16	FTSJ3	2.00E-09	0.18	Thyroid
ENSG00000108592.16	FTSJ3	3.40E-08	0.14	Muscle - Skeletal
ENSG00000108592.16	FTSJ3	5.20E-08	0.13	Esophagus - Mucosa
ENSG00000108592.16	FTSJ3	1.70E-07	0.14	Cells - Cultured fibroblasts
ENSG00000108592.16	FTSJ3	3.00E-07	0.2	Artery - Tibial
ENSG00000108592.16	FTSJ3	8.10E-07	0.17	Heart - Left Ventricle
ENSG00000108592.16	FTSJ3	9.50E-07	0.22	Colon - Transverse
ENSG00000108592.16	FTSJ3	0.0000032	0.18	Nerve - Tibial
ENSG00000108592.16	FTSJ3	0.0000036	0.17	Esophagus - Muscularis
ENSG00000108592.16	FTSJ3	0.0000049	0.21	Artery - Aorta
ENSG00000108592.16	FTSJ3	0.0000098	0.26	Pancreas
ENSG00000108592.16	FTSJ3	0.000011	0.13	Lung

ENSG00000108592.16	FTSJ3	0.000012	0.2	Esophagus - Gastroesophageal Junction
ENSG00000108592.16	FTSJ3	0.000015	0.13	Skin - Sun Exposed (Lower leg)
ENSG00000108592.16	FTSJ3	0.000028	0.2	Breast - Mammary Tissue
ENSG00000108592.16	FTSJ3	0.000042	0.16	Adipose - Visceral (Omentum)
ENSG00000108592.16	FTSJ3	0.00008	0.22	Prostate
ENSG00000108592.16	FTSJ3	0.0001	0.15	Adipose - Subcutaneous
ENSG00000108592.16	FTSJ3	0.00018	0.13	Skin - Not Sun Exposed (Suprapubic)
ENSG00000173826.14	KCNH6	1.30E-30	0.53	Lung
ENSG00000087191.12	PSMC5	0.000085	0.096	Colon - Transverse
ENSG00000240280.6	TCAM1P	0.000028	-0.22	Nerve - Tibial
ENSG00000240280.6	TCAM1P	0.00023	-0.17	Adipose - Subcutaneous

Supplementary Table 5. Single-tissue eQTLs for rs442495 downloaded from GTExPortal

Jan. 7th 2020

Gencode ID	Gene Symbol	P-Value	Normalized Effect Size	Tissue
ENSG00000137845.14	ADAM10	6.00E-14	0.21	Esophagus - Mucosa
ENSG00000137845.14	ADAM10	5.80E-10	0.18	Skin - Sun Exposed (Lower leg)
ENSG00000137845.14	ADAM10	2.40E-07	0.1	Muscle - Skeletal
ENSG00000137845.14	ADAM10	0.0000018	0.14	Skin - Not Sun Exposed (Suprapubic)
ENSG00000137845.14	ADAM10	0.0000026	-0.1	Nerve - Tibial
ENSG00000128923.10	MINDY2	7.60E-13	-0.21	Artery - Tibial
ENSG00000128923.10	MINDY2	1.90E-07	-0.098	Nerve - Tibial
ENSG00000128923.10	MINDY2	2.10E-07	-0.2	Artery - Aorta
ENSG00000128923.10	MINDY2	4.00E-07	0.08	Whole Blood
ENSG00000259353.1	RP11-30K9.5	0.000016	-0.25	Cells - Cultured fibroblasts
ENSG00000245975.2	RP11-30K9.6	1.80E-08	0.25	Thyroid
ENSG00000245975.2	RP11-30K9.6	0.0000054	0.32	Brain - Hypothalamus
ENSG00000245975.2	RP11-30K9.6	0.0000054	0.24	Pituitary
ENSG00000259250.1	RP11-50C13.1	0.000043	-0.18	Nerve - Tibial

Supplementary Table 6. Single-tissue eQTLs for rs4575098 downloaded from GTExPortal Jan. 7th 2020

Gencode ID	Gene Symbol	P-Value	Normalized Effect Size	Tissue
ENSG00000158859.9	ADAMTS4	2.30E-08	-0.28	Cells - Cultured fibroblasts
ENSG00000158850.14	B4GALT3	1.10E-14	-0.41	Cells - Cultured fibroblasts
ENSG00000158850.14	B4GALT3	6.00E-09	-0.18	Adipose - Subcutaneous
ENSG00000158850.14	B4GALT3	5.20E-08	-0.15	Skin - Sun Exposed (Lower leg)
ENSG00000158850.14	B4GALT3	1.40E-07	-0.21	Breast - Mammary Tissue
ENSG00000158850.14	B4GALT3	1.40E-06	-0.13	Nerve - Tibial
ENSG00000158850.14	B4GALT3	4.00E-06	-0.13	Skin - Not Sun Exposed (Suprapubic)
ENSG00000158869.10	FCER1G	3.40E-10	-0.13	Adipose - Subcutaneous
ENSG00000158869.10	FCER1G	1.20E-07	-0.15	Muscle - Skeletal
ENSG00000158869.10	FCER1G	1.80E-06	-0.12	Esophagus - Muscularis
ENSG00000158869.10	FCER1G	1.90E-06	-0.1	Adipose - Visceral (Omentum)
ENSG00000158869.10	FCER1G	2.80E-06	-0.078	Whole Blood
ENSG00000158869.10	FCER1G	5.70E-06	-0.12	Nerve - Tibial
ENSG00000158869.10	FCER1G	6.80E-06	-0.14	Colon - Transverse
ENSG00000158869.10	FCER1G	2.90E-05	-0.1	Lung
ENSG00000225217.1	HSPA7	1.60E-04	-0.21	Skin - Sun Exposed (Lower leg)
ENSG00000158864.12	NDUFS2	4.80E-07	-0.25	Brain - Cerebellum
ENSG00000158864.12	NDUFS2	8.80E-07	-0.13	Adipose - Subcutaneous
ENSG00000158864.12	NDUFS2	1.20E-06	-0.16	Nerve - Tibial
ENSG00000158864.12	NDUFS2	1.90E-06	-0.16	Skin - Not Sun Exposed (Suprapubic)
ENSG00000158864.12	NDUFS2	3.00E-06	-0.092	Whole Blood
ENSG00000158864.12	NDUFS2	3.30E-06	-0.12	Skin - Sun Exposed (Lower leg)
ENSG00000158864.12	NDUFS2	5.70E-06	-0.18	Breast - Mammary Tissue
ENSG00000158864.12	NDUFS2	1.30E-05	-0.15	Artery - Tibial
ENSG00000158864.12	NDUFS2	3.50E-05	-0.18	Artery - Aorta
ENSG00000158864.12	NDUFS2	4.90E-05	-0.23	Brain - Caudate (basal ganglia)

Supplementary Table 7. Single-tissue eQTLs for rs10496731 downloaded from GTExPortal Feb. 28th 2019

Gencode ID	Gene Symbol	P-Value	Normalized Effect Size	Tissue
ENSG00000224043.3	AC016725.4	1.30E-05	0.21	Testis
ENSG00000224043.3	AC016725.4	2.70E-05	-0.42	Pituitary
ENSG00000082258.8	CCNT2	1.20E-08	0.21	Testis
ENSG00000082258.8	CCNT2	2.30E-08	0.15	Thyroid
ENSG00000082258.8	CCNT2	1.60E-07	0.14	Nerve - Tibial
ENSG00000082258.8	CCNT2	2.50E-07	0.14	Skin - Not Sun Exposed (Suprapubic)
ENSG00000082258.8	CCNT2	4.50E-07	0.27	Pituitary
ENSG00000082258.8	CCNT2	9.80E-06	0.14	Esophagus - Muscularis
ENSG00000082258.8	CCNT2	2.20E-05	0.1	Artery - Tibial
ENSG00000082258.8	CCNT2	2.80E-05	0.16	Heart - Left Ventricle
ENSG00000082258.8	CCNT2	5.80E-05	0.11	Lung
ENSG00000152128.13	TMEM163	4.30E-10	0.35	Testis
ENSG00000152128.13	TMEM163	4.50E-10	-0.41	Esophagus - Muscularis
ENSG00000152128.13	TMEM163	2.20E-06	-0.26	Pituitary
ENSG00000152128.13	TMEM163	5.90E-05	-0.22	Esophagus - Mucosa
ENSG00000144224.12	UBXN4	5.60E-06	-0.12	Skin - Not Sun Exposed (Suprapubic)

Supplementary Table 8. Single-tissue eQTLs for rs11642612 downloaded from GTExPortal Feb. 28th 2019

Excel Document

Supplementary Table 9. Single-tissue eQTLs for rs12595082 downloaded from GTExPortal Feb. 28th 2019

Gencode ID	Gene Symbol	P-Value	Normalized Effect Size	Tissue
ENSG00000138600.5	SPPL2A	9.70E-14	0.38	Adipose - Subcutaneous
ENSG00000081014.6	AP4E1	0.000057	0.18	Adipose - Subcutaneous
ENSG00000138600.5	SPPL2A	3.00E-10	0.25	Adipose - Visceral (Omentum)
ENSG00000138600.5	SPPL2A	2.10E-07	0.31	Adrenal Gland
ENSG00000138600.5	SPPL2A	4.60E-07	0.24	Artery - Aorta
ENSG00000138600.5	SPPL2A	6.40E-09	0.35	Artery - Coronary
ENSG00000138600.5	SPPL2A	2.90E-12	0.32	Artery - Tibial
ENSG00000138592.9	USP8	2.20E-08	0.19	Artery - Tibial
ENSG00000138600.5	SPPL2A	0.0000026	0.33	Brain - Frontal Cortex (BA9)
ENSG00000140287.6	HDC	0.0000082	-0.51	Brain - Hippocampus
ENSG00000138600.5	SPPL2A	0.000017	0.3	Brain - Nucleus accumbens (basal ganglia)
ENSG00000138600.5	SPPL2A	2.40E-23	0.5	Cells - Transformed fibroblasts
ENSG00000138600.5	SPPL2A	3.50E-07	0.25	Colon - Sigmoid
ENSG00000138600.5	SPPL2A	0.000014	0.16	Colon - Transverse
ENSG00000138600.5	SPPL2A	0.000031	0.19	Esophagus - Mucosa
ENSG00000138600.5	SPPL2A	3.20E-13	0.28	Esophagus - Muscularis
ENSG00000138592.9	USP8	1.70E-09	0.17	Esophagus - Muscularis
ENSG00000138600.5	SPPL2A	5.80E-11	0.27	Heart - Atrial Appendage
ENSG00000138600.5	SPPL2A	7.40E-08	0.16	Heart - Left Ventricle
ENSG00000138600.5	SPPL2A	3.90E-14	0.28	Lung
ENSG00000138600.5	SPPL2A	0.0000058	0.54	Minor Salivary Gland
ENSG00000138600.5	SPPL2A	3.80E-14	0.25	Muscle - Skeletal
ENSG00000241130.1	RP11-507J18.1	5.30E-12	-0.38	Muscle - Skeletal
ENSG00000138600.5	SPPL2A	1.70E-15	0.38	Nerve - Tibial
ENSG00000138600.5	SPPL2A	0.000013	0.37	Pituitary
ENSG00000138600.5	SPPL2A	0.000091	0.16	Skin - Sun Exposed (Lower leg)
ENSG00000138600.5	SPPL2A	0.0000089	0.35	Testis
ENSG00000138600.5	SPPL2A	3.80E-11	0.25	Thyroid
ENSG00000138592.9	USP8	0.000046	0.16	Thyroid

Supplementary Table 10. List of approximate conditional analyses

Trait	Lead SNP	Chr	Position GRCh37	Conditional SNPs
BMI	rs359539	3	155282432	rs4680176
T2D	rs10920254	1	201767474	rs2820311
CHD	rs28394864	17	47450775	rs4643373, rs1294468
T2D	rs4351	17	61569732	rs7210095
TG	rs4311	17	61560763	rs894407
UBXN4 eQTL in Not Sun Exposed Skin	rs10496731	2	135597628	rs23207727
WHRadjBMI	rs605928	15	59046163	rs112075474
AD	rs605928	15	59046163	rs112075474
AD	rs4308	17	61559625	rs4311
AD	rs4308	17	61559625	rs4324
AD	rs4324	17	61563171	rs4308
ACE eQTL in Lung	rs4308	17	61559625	rs4324
ACE eQTL in Lung	rs4324	17	61563171	rs4308
ACE eQTL in Cerebellum	rs4308	17	61559625	rs4342
ACE eQTL in Cerebellum	rs4342	17	61565998	rs4308
ACE eQTL in Cerebellum	rs4308	17	61559625	rs4342
ACE eQTL in Cerebellum	rs4342	17	61565998	rs4308
T2D	rs4324	17	61563171	rs4308, rs7210095
T2D	rs4308	17	61559625	rs4342, rs7210095

Supplementary Table 11. AD-centric bivariate analysis single-tissue-eQTL results

Cardiometabolic Trait	Locus Name	Lead SNP	Effect Allele/ Other Allele	Direction of Effect AD/CM	Bivariate P-value	eGene(s)	Tissue	Conditional Posterior Probability of Colocalization	Direction of Expression of eQTL
DBP	*ADAM10*	rs442495	T/C	+/+	1.98e-10	MINDY2	Tibial Nerve	0.90	+
WHRadj BMI	*ADAMTS4*	rs4575098	A/G	+/+	4.45e-13	NDUFS2	Whole Blood	1.00	-
WHRadj BMI	*ADAMTS4*	rs4575098	A/G	+/+	4.45e-13	NDUFS2	Aorta Artery	0.98	-
WHRadj BMI	*ADAMTS4*	rs4575098	A/G	+/+	4.45e-13	NDUFS2	Tibial Artery	0.93	-
WHRadj BMI	*ADAMTS4*	rs4575098	A/G	+/+	4.45e-13	NDUFS2	Skin - Not Sun Exposed	0.88	-
WHRadj BMI	*ADAMTS4*	rs4575098	A/G	+/+	4.45e-13	NDUFS2	Brain - Basal Ganglia	0.83	-
WHRadj BMI	*ADAMTS4*	rs4575098	A/G	+/+	4.45e-13	FCER1G	Tibial Nerve	0.81	-
WHRadj BMI	*ADAMTS4*	rs4575098	A/G	+/+	4.45e-13	NDUFS2	Subcutaneous Adipose	0.81	-
DBP	*ACE*	rs4308	G/A	+/-	7.05e-15	ACE	Skin - Not Sun Exposed	0.99	-
DBP	*ACE*	rs4308	G/A	+/-	7.05e-15	ACE	Kidney Cortex	0.99	+
DBP	*ACE*	rs4308	G/A	+/-	7.05e-15	ACE	Transverse Colon	0.98	+
DBP	*ACE*	rs4308	G/A	+/-	7.05e-15	DCAF7	Lung	0.98	+

DBP	*ACE*	rs4308	G/A	+/-	7.05e-15	ACE	Subcutaneous Adipose	0.97	-
DBP	*ACE*	rs4308	G/A	+/-	7.05e-15	ACE	Visceral Adipose	0.97	-
DBP	*ACE*	rs4308	G/A	+/-	7.05e-15	ACE	Skin - Sun Exposed	0.97	-
DBP	*ACE*	rs4308	G/A	+/-	7.05e-15	ACE	Brain – Frontal Cortex	0.94	-
DBP	*ACE*	rs4308	G/A	+/-	7.05e-15	ACE	Adrenal Gland	0.93	-
DBP	*ACE*	rs4308	G/A	+/-	7.05e-15	ACE	Tibial Nerve	0.90	-
DBP	*ACE*	rs4308	G/A	+/-	7.05e-15	ACE	Aorta Artery	0.89	-
DBP	*ACE*	rs4308	G/A	+/-	7.05e-15	ACE	Lung	0.89	+

Direction of effect first position is the direction of effect of the effect allele on AD and the second position is the direction of effect of the effect allele on the cardiometabolic trait; Conditional Posterior Probability of Colocalization, PP4/ (PP3 + PP4) from the results of the single-tissue-eQTL colocalization analysis

Table S12. Locus discovery analysis single-tissue-eQTL results
Excel Document

Supplemental Table 13. Human Splice Finder 3 results for rs144867634

Sequence Position	cDNA Position	Splice site type	Motif	New splice site	Wild Type	Mutant	Variation	(%)
227	127	Acceptor	TATACATgtaagaa	tatacgtgtaagAA	72.07	72.02	NA	-0.07
231	131	Donor	CATgtaaga	CGTgtaaga	86.8	81.94	142	-5.6

Supplemental Table 14. AVISPA results on rs144867634 A allele

AVISPA Analysis	False Positive Rate	Rank	Sensitivity
Exon is alternative vs constitutively spliced	0.016	0.048	0.399
Splicing is altered in central nervous system tissues	0.091	0.077	0.569
Splicing is altered in muscle tissue	0.085	0.067	0.573
Splicing is altered in embryo tissue	0.114	0.125	0.682
Splicing is altered in digestive tissues	0.043	0.070	0.567

FPR, false positive rate based on the data used to train the AVISPA model; Rank, the fraction of samples in the reference data that have a score at least as high as the query; Sensitivity, based on the data used to train the AVISPA model.

Supplemental Table 15. AVISPA results on rs144867634 G allele

AVISPA Analysis	False Positive Rate	Rank	Sensitivity
Exon is alternative vs constitutively spliced	0.002	0.008	0.136
Splicing is altered in central nervous system tissues	0.012	0.008	0.219
Splicing is altered in muscle tissue	0.044	0.020	0.285
Splicing is altered in embryo tissue	0.070	0.062	0.527
Splicing is altered in digestive tissues	0.022	0.013	0.190

FPR, false positive rate based on the data used to train the AVISPA model; Rank, the fraction of samples in the reference data that have a score at least as high as the query; Sensitivity, based on the data used to train the AVISPA model.

Supplemental References:

1. Purcell S, Neale B, Todd-Brown K, Thomas L, Ferreira MAR, Bender D, et al. PLINK: A tool set for whole-genome association and population-based linkage analyses. Am J Hum Genet. 2007;81(3):559–75.
2. Zhao W, Rasheed A, Tikkanen E, Lee JJ, Butterworth AS, Howson JMM, et al. Identification of new susceptibility loci for type 2 diabetes and shared etiological pathways with coronary heart disease. Nat Genet. 2017 Oct 1;49(10):1450–7.
3. Ray D, Boehnke M. Methods for meta-analysis of multiple traits using GWAS summary statistics. Genet Epidemiol [Internet]. 2018 [cited 2019 Aug 21];42(2):134–45. Available from: http://www.ncbi.nlm.nih.gov/pubmed/29226385
4. Siewert KM, Voight BF. Bivariate Genome-Wide Association Scan Identifies 6 Novel Loci Associated With Lipid Levels and Coronary Artery Disease. Circ Genomic Precis Med [Internet]. 2018 Dec [cited 2019 Mar 8];11(12). Available from: https://www.ahajournals.org/doi/10.1161/CIRCGEN.118.002239
5. Hemani G, Zheng J, Elsworth B, Wade KH, Haberland V, Baird D, et al. The MR-Base platform supports systematic causal inference across the human phenome. Elife [Internet]. 2018 May 30 [cited 2018 Aug 29];7. Available from: https://elifesciences.org/articles/34408
6. Altshuler DM, Durbin RM, Abecasis GR, Bentley DR, Chakravarti A, Clark AG, et al. An integrated map of genetic variation from 1,092 human genomes. Nature. 2012 Nov 1;491(7422):56–65.
7. Giambartolomei C, Zhenli Liu J, Zhang W, Hauberg M, Shi H, Boocock J, et al. A Bayesian framework for multiple trait colocalization from summary association statistics. Berger B, editor. Bioinformatics [Internet]. 2018 Aug 1 [cited 2019 Apr 1];34(15):2538–45. Available from: http://www.ncbi.nlm.nih.gov/pubmed/29579179
8. Xin J, Mark A, Afrasiabi C, Tsueng G, Juchler M, Gopal N, et al. High-performance web

services for querying gene and variant annotation. Genome Biol [Internet]. 2016 May 6 [cited 2020 Mar 19];17(1):91. Available from: http://genomebiology.biomedcentral.com/articles/10.1186/s13059-016-0953-9

9. Rentzsch P, Witten D, Cooper GM, Shendure J, Kircher M. CADD: Predicting the deleteriousness of variants throughout the human genome. Nucleic Acids Res. 2019 Jan 8;47(D1):D886–94.

10. Desmet FO, Hamroun D, Lalande M, Collod-Bëroud G, Claustres M, Béroud C. Human Splicing Finder: An online bioinformatics tool to predict splicing signals. Nucleic Acids Res. 2009;37(9).

11. Barash Y, Vaquero-Garcia J, González-Vallinas J, Xiong HY, Gao W, Lee LJ, et al. AVISPA: A web tool for the prediction and analysis of alternative splicing. Genome Biol [Internet]. 2013 Oct 24 [cited 2020 Mar 19];14(10):R114. Available from: http://www.ncbi.nlm.nih.gov/pubmed/24156756
